# Cytotoxic Triterpene Glycosides from Mexican Sea Cucumber *Holothuria inornata*

**DOI:** 10.1021/acs.jnatprod.5c00716

**Published:** 2025-09-22

**Authors:** Esteban López-Sampedro, Roberto Arreguin-Espinosa, Ana M. Simonet

**Affiliations:** † Posgrado en Ciencias del Mar y Limnología, Universidad Nacional Autónoma de México, Av. Universidad 3000, Ciudad Universitaria Coyoacán, C.P. 04510 Ciudad de México, México; ‡ Department of Organic Chemistry, Institute of Biomolecules (INBIO), Campus de Excelencia Internacional (ceiA3), School of Science, University of Cadiz, 11510 Puerto Real, Cadiz, Spain; § Departamento de Química de Biomacromoléculas, Instituto de Química, Universidad Nacional Autónoma de México, Av. Universidad 3000, Circuito Exterior s/n, Coyoacán, Ciudad Universitaria, México City 04510, México

## Abstract

Sea cucumbers (Holothuriidae) are highly valued in traditional medicine and are recognized for their diverse bioactivities, which are largely attributed to their rich triterpene glycoside content. Given that *Holothuria inornata* was chemically uncharacterized, this study describes the bioassay-guided isolation of these compounds. Nine sulfated saponins were purified, and their structures were elucidated using UPLC-QTOF/HR-ESI-MS^*n*^ and comprehensive NMR spectroscopy. Six new compounds were identified, including inornatosides A–C (**1**–**3**) and 22*R*-holothurin B (**6**), all possessing a holostane-type aglycone, alongside inornatosides D (**4**) and E (**5**), which feature unique aglycones. Cytotoxic assays revealed that inornatoside B (**2**) exhibited potent cytotoxic activity against human mammary adenocarcinoma (MCF-7) and human lung adenocarcinoma (SKLU-1) cell lines, with IC_50_ values between 0.47 and 0.50 μM surpassing the efficacy of the positive control. These findings not only expand the chemical diversity of holothurins but also position *H. inornata* as a promising source of anticancer compounds.

## Graphical abstract

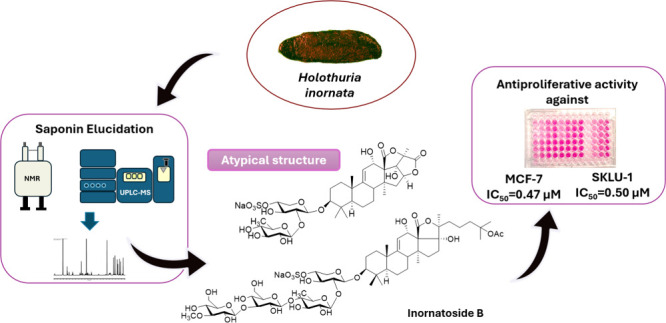

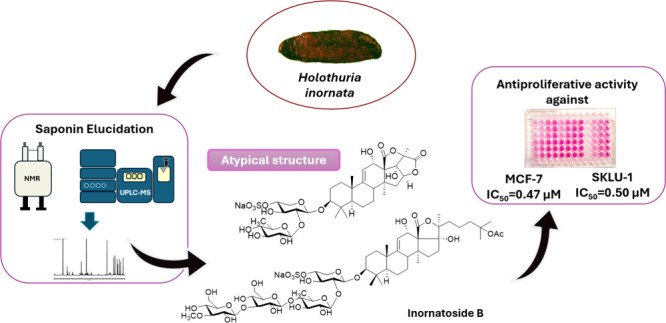

Holothuroids, commonly known as “sea cucumbers,” are marine invertebrates belonging to the class Holothuroidea within the phylum Echinodermata.^1^ With over 1,770 species described, most sea cucumbers are benthic organisms that play significant ecological roles and are increasingly valued for their bioactive compounds.^2^ Among these compounds, saponins stand out as a major class of natural products in sea cucumbers, acting as a crucial chemical defense against predation.^3^ These compounds are found in high concentrations within organs critical for defense, including those involved in evisceration, such as the Cuvierian tubules or intestines.^4^

Sea cucumbers have long been utilized as a food resource, particularly in Asian countries, where they are consumed as a luxury delicacy (bêche-de-mer) and are believed to possess healing, nutritious, and aphrodisiac properties.^5,6^ Nutritionally, sea cucumbers are characterized by a high protein content, notably including amino acids such as lysine, arginine, and tryptophan, coupled with a lower fat content compared to many other food sources.^7^

In East Asian traditional medicine, beyond their culinary applications,^8^ sea cucumbers have a long history of use in treating conditions such as asthma, hypertension, rheumatism,^9^ anemia,^10^ and sinus congestion.^11^ They have also been reported as effective in healing various internal and external wounds. The tissue repair capability of sea cucumbers has been associated with its high eicosapentaenoic acid (EPA) content.^12^ Their medicinal potential is largely attributed to their rich diversity of bioactive molecules, including lipids, chondroitin sulfates, glycosaminoglycans, sulfated polysaccharides,^13^ and saponins.^14^ Notably, saponins from sea cucumbers have demonstrated significant anticancer properties, inhibition of hyperlipidemia and fatty liver, and regulation of blood glucose concentrations, among other effects.^15^ The membranotropic and membranolytic activities of saponins (e.g., frondosides,^16^ stichoposides, cucumariosides,^17^ or holothurins^18^), along with their ability to induce cytotoxicity and apoptosis, have been extensively studied. These compounds have been shown to inhibit the proliferation of various cancer cell lines, including breast, colorectal, gastric, and lung cancers.^15^

Mexico’s coasts exhibit a remarkable diversity of sea cucumbers,^19^ species recognized for their value since antiquity. Evidence from as early as the 14th century shows that Mexica priests in Tenochtitlan included five species of sea cucumbers among the offerings buried within religious structures.^20^ Despite their limited consumption by the contemporary Mexican populace, these organisms face significant illegal exploitation driven by the high economic returns they command in the Asian market.^21^

Beyond their economic importance, sea cucumbers play a vital ecological role as benthic ecosystem engineers, integral to processing organic matter within the detrital food web. To mitigate the pressure on wild populations and facilitate the recovery of depleted stocks, aquaculture^22^ emerges as a crucial tool for conservation.

*Holothuria inornata,* Semper 1868, Holothuriidae, previously considered the same species as *H. kefersteinii*,^23^ is distributed in the Pacific Ocean. Prior research on this species has primarily focused on its basic biology and ecology, including reproductive cycles and the development of *H. inornata* juveniles fed an algal diet.^24^ Despite this, the saponin content and potential bioactivities of *H. inornata* have remained uninvestigated, leaving a significant gap in our understanding of its chemical composition and potential applications. This study focuses on the bioassay-guided isolation of these saponins. The active fraction was analyzed by UPLC-QTOF/MS^*n*^ to confirm the presence of saponins, from which nine sulfated saponins were subsequently purified and structurally elucidated using comprehensive NMR spectroscopy. Six of these compounds are described for the first time, highlighting the structures of inornatosides D (**4**) and E (**5**), which feature unusual aglycones (Chart 1). Furthermore, the cytotoxicity of the major holothurins was evaluated across several cancer cell lines. Notably, inornatoside B (**2**) demonstrated promising and stronger cytotoxic activity against human mammary adenocarcinoma (MCF-7) and human lung adenocarcinoma (SKLU) cell lines compared with the positive control.

**Chart 1:**
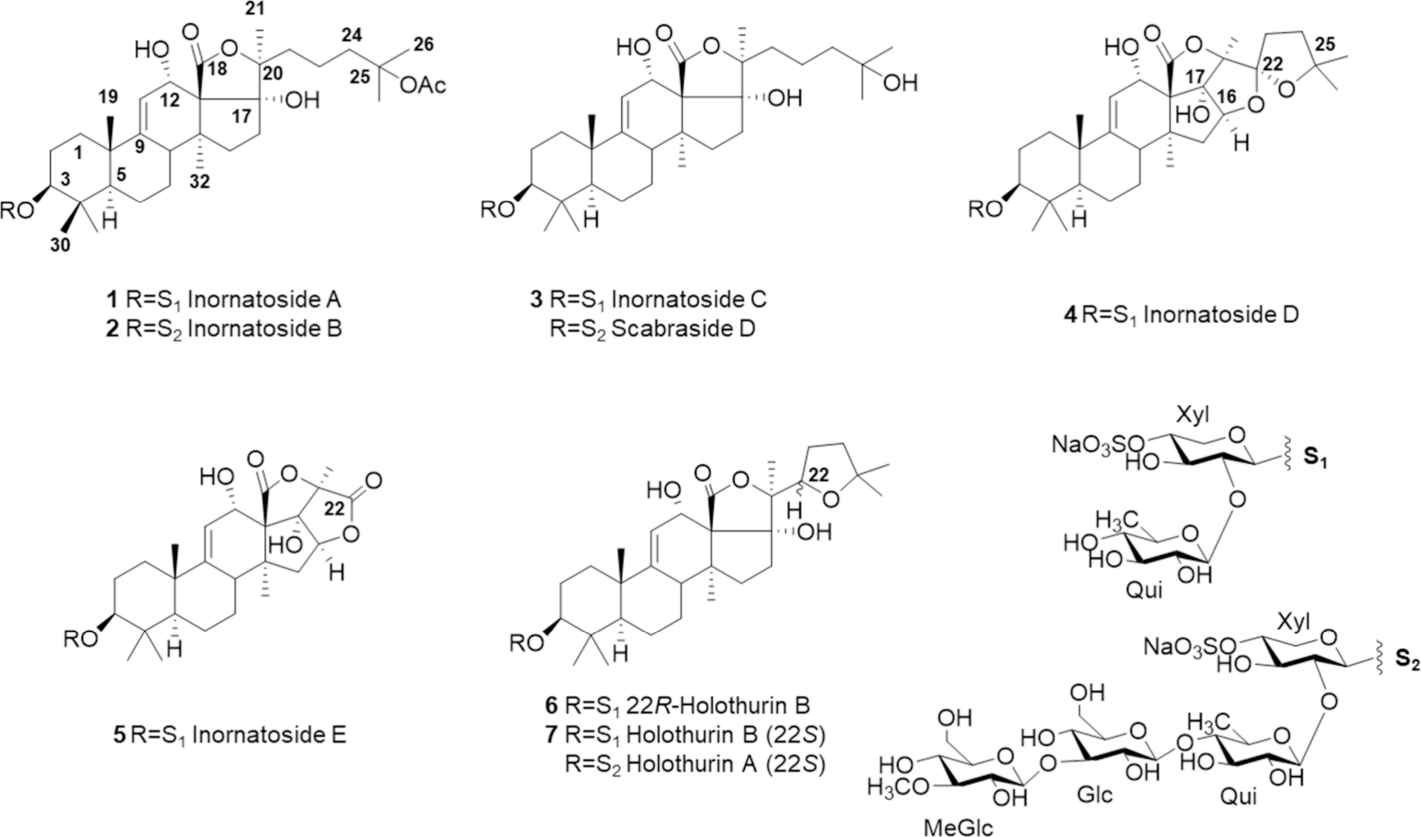
Saponins isolated from *Holothuria inornata*

By elucidating the saponin profile of *H. inornata*, this study not only contributes to understanding its chemical diversity but also highlights its potential as a valuable resource for food and medicinal applications. This work aligns with the growing interest in marine organisms as sustainable sources of bioactive compounds.

## Results and Discussion

### Bioassay-Guided Separation of Cytotoxic Saponins

Extracts obtained from the body wall of *Holothuria inornata* (Mazatlán, Mexico) were subjected to a partitioning and desalting process to yield a saponin extract. This extract then underwent reverse-phase column chromatography with a stepped water–acetone gradient to obtain a saponin enriched fraction. Both the crude saponin extract and the enriched fraction were evaluated for their cytotoxic activity based on their growth inhibition effect on the cancer cell lines human glioblastoma (U251), human prostatic adenocarcinoma (PC-3), human chronic myelogenous leukemia (K562), human colorectal adenocarcinoma (HCT-15), human mammary adenocarcinoma (MCF-7), and human lung adenocarcinoma (SKLU-1) using the SRB method at concentration of 25 μg/mL. Results showed that crude saponin extract exhibited moderate growth inhibition across the tested cell lines, whereas the enriched fraction demonstrated high inhibition (Figure 1).

**Figure 1:**
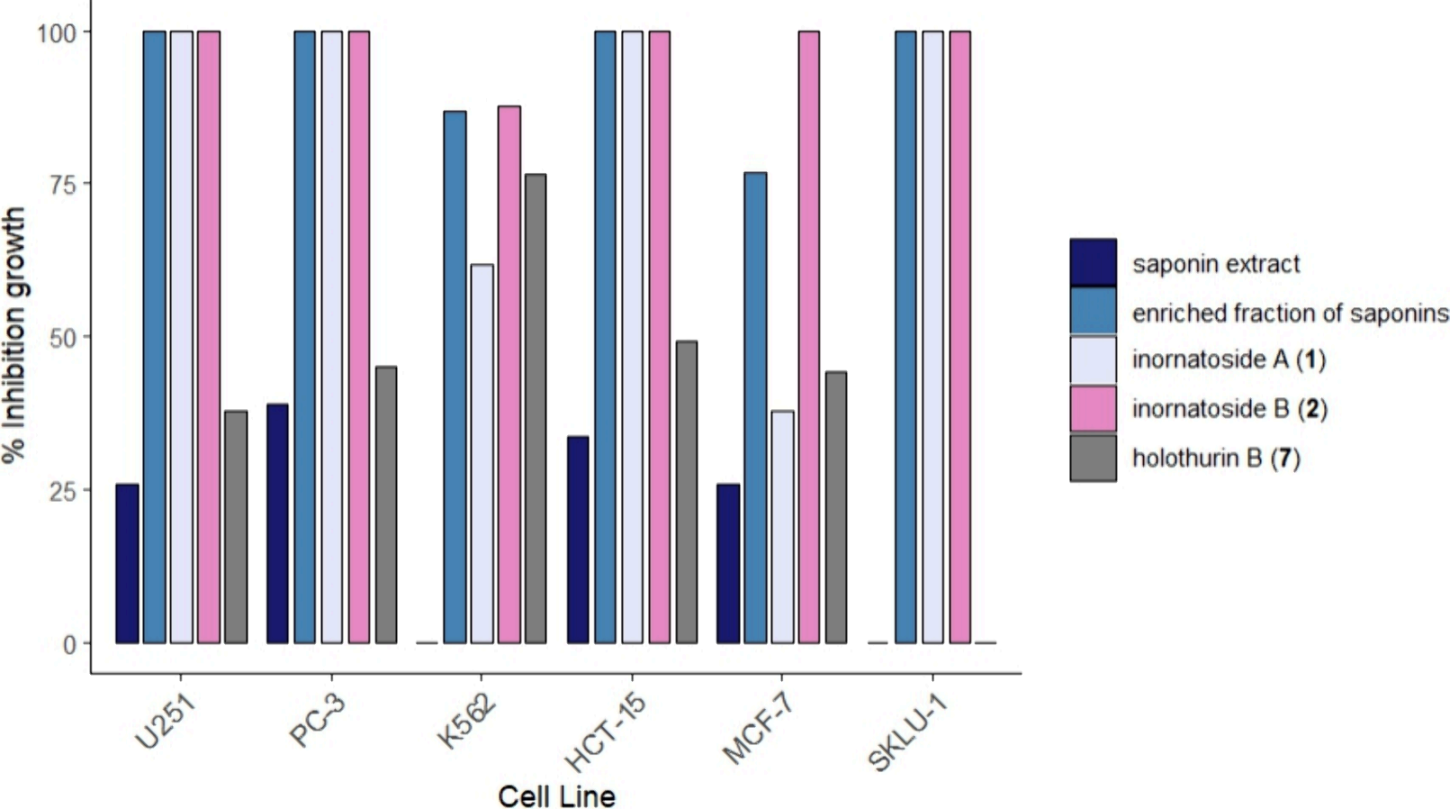
Saponin extract, enriched fraction, and major saponins inhibited the proliferation of six cancer cell lines using the SRB method at concentration of 25 μg/mL.

UPLC-ESI-MS analysis of this fraction revealed the characteristic ions of four major holothurins (Figure 2). Subsequent sequential separations led to the isolation of nine saponins. Six of these compounds are reported herein for the first time, designated as inornatosides A–E (**1**–**5**) and 22*R*-holothurin B (**6**). The remaining isolated saponins were identified as scabraside D,^25^ holothurin B^26^ (**7**), and holothurin A.^27,28^

**Figure 2:**
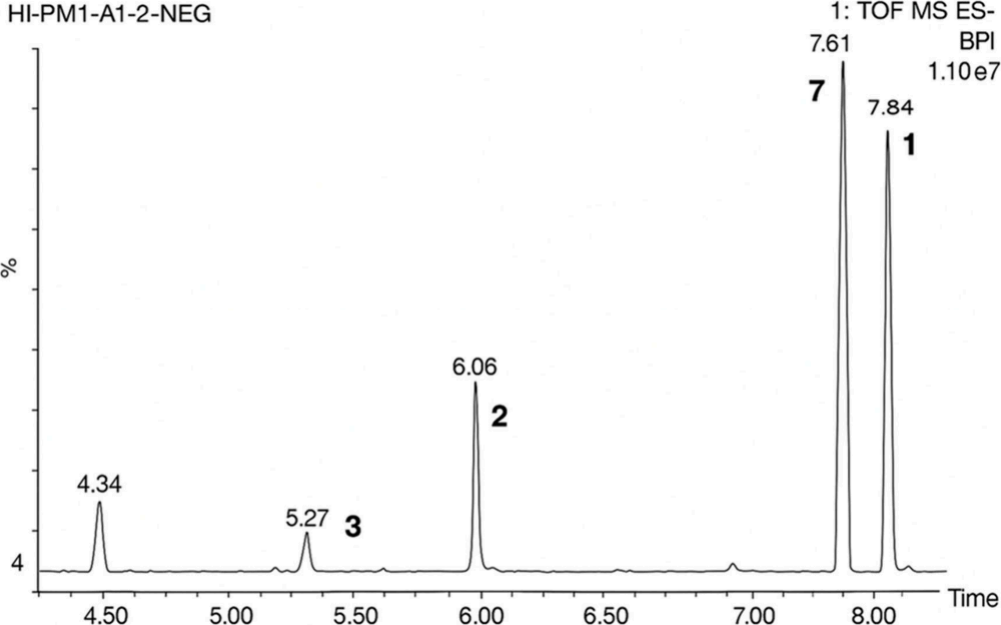
UPLC-ESI-MS analysis of saponin enriched fraction with major saponins assigned.

### Structure Elucidation of Isolated Holothurins

The structure elucidation of the isolated saponins was achieved through the application of one- and two-dimensional NMR spectroscopic techniques. Complete assignment of all ^1^H and ^13^C NMR signals for each saponin was accomplished, as detailed in Tables 1, 2, and 3. Notably, one-dimensional TOCSY and Nuclear Overhauser Effect Spectroscopy (NOESY) experiments were employed via selective excitation of the anomeric proton signals of each monosaccharide unit. The NMR signals corresponding to the sugar moieties and the A and B rings of the isolated saponins exhibited superimposable characteristics with those obtained for holothurin A^29^ or B (**7**)^30^ (Figures S31 and S32, Tables 1–3). Consequently, the isolated saponins can be classified into two distinct groups based on their sugar chains. To confirm the absolute configuration of the monosaccharides in both chains and considering the limited availability of minor saponins, hydrolysis of fraction F was proposed. This fraction contains saponins with two possible sugar chains in a 1:1 ratio. Subsequent purification of the monosaccharides and measurement of their optical rotation verified the absolute configuration of the chains to be consistent with previous descriptions.

**Table 1:** ^13^C and ^1^H NMR Data (*J* in Hz) for the Aglycone Moieties of Compounds **1** and **3**–**6** (Pyridine-*d*_*5*_)­^a^

	Inornatoside A (**1**)		Inornatoside C (**3**)		Inornatoside D (**4**)		Inornatoside E (**5**)		22*R*-Holothurin B (**6**)	
	δ_C_	δ_H_	δ_C_	δ_H_	δ_C_	δ_H_	δ_C_	δ_H_	δ_C_	δ_H_
1_ax_	36.4	1.40 ddd (13, 13, 3)	36.4	1.39 ddd (13, 13, 4)	36.4	1.35 ddd (13, 13, 4)	36.3	1.34 ddd (13, 13, 3)	36.5	1.38 ddd (13, 13, 3)
1_eq_		1.82 brd (13)		1.82 dd (13, 4)		1.77 brd (13)		1.74 ddd (13, 3, 3)		1.80 brd (13)
2_ax_	27.1	1.90 brddd (13, 13, 13)	27.1	1.91 dddd (14, 14, 12, 3)	27.1	1.88 brddd (13, 13, 13)	27.1	1.88 dddd (14, 13, 13, 3)	27.1	1.89 dddd (13, 13, 13, 3)
2_eq_		2.07 brdd (13, 4)		2.09 brdd (14, 4)		2.07 brdd (13, 4)		2.08 brdd (14, 4)		2.08 brdd (13, 3)
3	88.8	3.14 dd (12, 4)	88.8	3.15 dd (12, 4)	88.7	3.12 dd (12, 4)	88.6	3.13 dd (12, 4)	88.8	3.14 dd (12, 4)
4	40.2		40.2		40.2		40.1		40.2	
5	52.8	0.99 dd (11, 2)	52.8	0.99 dd (12, 2)	52.7	0.95 dd (12, 2)	52.6	0.93 dd (12, 2)	52.8	0.98 brd (11)
6_ax_	21.3	1.55 brddd (13, 12, 11)	21.3	1.55 brddd (13, 13, 12)	21.2	1.50 dddd (13, 13, 12, 3)	21.0	1.49 dddd (13, 13, 13, 3)	21.3	1.54 brddd (12, 12, 11)
6_ec_		1.74 o		1.73 o		1.69 brd (13)		1.69 brd (13)		1.73 o
7_ax_	28.4	1.48 dddd (13, 12, 12, 4)	28.4	1.49 dddd (13, 13, 13, 4)	28.6	1.40 dddd (13, 13, 13, 4)	28.3	1.34 dddd (13, 13, 13, 4)	28.4	1.48 dddd (13, 12, 12, 4)
7_eq_		1.74 o		1.74 o		1.63 m		1.62 brddd (13, 6, 3)		1.73 o
8	41.0	3.34 brdd (13, 5)	40.9	3.35 brdd (13, 5)	40.8	3.30 brdd (13, 6)	40.3	2.98 dd (13, 6)	40.8	3.35 brdd (12, 5)
9	154.2		154.1		154.0		153.5		154.2	
10	39.8		39.8		39.9		39.9		39.8	
11	115.7	5.58 dd (5, 2)	115.7	5.58 dd (6, 2)	115.3	5.60 brd (6)	115.3	5.59 dd (6, 2)	115.7	5.55 dd (6, 2)
12	71.5	4.97 brd (5)	71.4	4.97 brd (6)	70.2	4.91 brd (6)	69.4	4.90 brs	71.4	4.97 brs
13	58.6		58.7		56.5		56.3		58.3	
14	46.5		46.5		49.7		47.8		45.8	
15_a_	36.8	1.40 ddd (12, 12, 9)	36.8	1.39 ddd (12, 12, 9)	45.5	1.56 dd (13, 6)	43.5	1.30 dd (13, 9)	36.8	1.38 ddd (13, 12, 9)
15_b_		1.82 dd (12, 9)		1.80 dd (12, 9)		2.39 dd (13, 8)		2.38 dd (13, 8)		1.80 dd (12, 8)
16_a_	36.0	2.66 dd (14, 9)	36.0	2.68 dd (14, 9)	90.3	5.01 dd (8, 6)	91.8	5.22 dd (9, 8)	35.5	2.58 dd (15, 9)
16_b_		2.34 ddd (14, 12, 9)		2.32 ddd (14, 12, 9)						2.18 ddd (15, 13, 8)
17	89.4		89.4		94.7		83.2		88.5	
18	174.8		174.9		174.2		172.9		174.9	
19	22.7	1.36 s	22.7	1.36 s	22.5	1.34 s	22.3	1.29 s	22.7	1.35 s
20	87.1		87.4		89.8		90.0		88.7	
21	23.1	1.76 s	23.1	1.76 s	16.0	1.70 s	16.6	1.80 s	17.5	1.77 s
22	39.0	2H, 1.90 t (8)	39.5	2H, 1.95 (o)	119.7		173.6		82.3	4.43 dd (8, 8)
23_a_	19.1	1.74 m	19.7	1.95 (o)	32.6	2.30 ddd (14, 9, 9)			27.9	2.21 dddd (14, 8, 7, 7)
23_b_		1.64 m		1.85 (o)		2.24 ddd (14, 8, 4)				2.06 dddd (14, 8, 8, 8)
24_a_	41.5	1.86 m	45.3	2H 1.67 (o)	37.2	1.90 ddd (12, 8, 9)			38.5	1.66 m
24_b_		1.81 m				1.61 (o)				1.61 m
25	82.1		69.5		83.9				81.2	
26	26.2	1.46 s	30.1	1.39 s	29.7	1.35 s			28.6	1.27 s
27	26.2	1.48 s	30.0	1.39 s	28.5	1.14 s			27.9	1.24 s
30	16.8	1.12 s	16.8	1.12 s	16.8	1.10 s	16.8	1.10 s	16.8	1.11 s
31	28.2	1.28 s	28.2	1.28 s	28.2	1.26 s	28.2	1.27 s	28.2	1.27 s
32	20.2	1.65 s	20.2	1.65 s	21.8	1.63 s	21.9	1.52 s	20.1	1.64 s
CH_3_CO	22.4	1.95 s								
CH_3_ CO	170.3									
OH-12		8.19 brs		8.19 brs		8.24 brs				8.37 d (4)
OH-17		7.41 s		7.42 s		7.95 s		8.40 s		7.48 s

a Assignments were confirmed by ^1^H–^1^H-COSY, 1D and 2D-TOCSY, HSQC, HSQC-TOCSY, and HMBC experiments. “o” indicates overlapped with other signals.

**Table 2:** ^13^C and ^1^H NMR Data (*J* in Hz) of the Sugar Chains of Compounds **1** and **3**–**6** (Pyridine-*d*_*5*_)­^a^

	Inornatoside A (**1**)		Inornatoside C (**3**)		Inornatoside D (**4**)		Inornatoside E (**5**)			22*R*-Holothurin B (**6**)
	δ_C_	δ_H_	δ_C_	δ_H_	δ_C_	δ_H_	δ_C_	δ_H_	δ_C_	δ_H_
1	105.4	4.70 d (7)	105.4	4.70 d (7)	105.4	4.70 d (7)	105.4	4.70 d (7)	105.4	4.70 d (7)
2	83.6	4.03 dd (7, 8)	83.5	4.08 dd (7, 9)	83.5	4.05 dd (7, 9)	83.5	4.07 dd (7, 9)	83.4	4.07 dd (7, 9)
3	75.6	4.30 dd (9, 9)	75.7	4.30 dd (9, 9)	75.9	4.33 dd (9, 9)	76.0	4.35 dd (9, 9)	75.9	4.34 dd (9, 9)
4	76.4	5.08 brs	76.1	5.12 (o)	75.9	5.13 ddd (9, 9, 5)	75.8	5.16 ddd (9, 9, 5)	76.0	5.15 ddd (9, 9, 5)
5_ax_	64.3	3.72 dd (12, 11)	64.4	3.74 dd (12, 9)	64.5	3.73 dd (12, 9)	64.6	3.72 dd (12, 9)	64.5	3.73 dd (12, 9)
5_ec_		4.75 dd (12, 4)		4.75 dd (12, 5)		4.75 dd (12, 5)		4.75 dd (12, 5)		4.74 dd (12, 5)
1′	106.1	5.14 d (8)	106.1	5.14 d (8)	106.1	5.13 d (8)	106.1	5.14 d (8)	106.1	5.13 d (8)
2′	77.1	4.03 dd (8, 9)	77.1	4.03 dd (8, 9)	77.1	4.02 dd (8, 9)	77.2	4.04 dd (8, 9)	77.2	4.03 dd (8, 9)
3′	77.9	4.12 dd (9, 9)	77.9	4.10 dd (9, 9)	77.9	4.09 dd (9, 9)	77.9	4.10 dd (9, 9)	77.9	4.10 dd (9, 9)
4′	76.8	3.70 dd (9, 9)	76.8	3.71 dd (9, 9)	76.8	3.70 dd (9, 9)	76.8	3.72 dd (9, 9)	76.8	3.70 dd (9, 9)
5′	73.6	3.76 o	73.6	3.75 dq (9, 6)	73.6	3.75 dq (9, 6)	73.6	3.76 dq (9, 6)	73.6	3.74 dq (9, 6)
6′	18.7	1.64 d (5)	18.7	1.66 d (6)	18.7	1.65 d (6)	18.8	1.67 d (6)	18.8	1.65 d (6)

a Assignments were confirmed by ^1^H–^1^H-COSY, 1D and 2D-TOCSY, NOESY, HSQC, HSQC-TOCSY, and HMBC experiments. “o” indicates overlapped with other signals.

**Table 3:** ^13^C and ^1^H NMR Data (*J* in Hz) for Inornatoside B (**2**) (Pyridine-*d*_5_)­^a^

	δ_C_	δ_H_		δ_C_	δ_H_
1_ax_	36.4	1.39 ddd (13, 13, 3)	Sugar chain		
1_eq_		1.81 ddd (13, 3, 3)	1	105.4	4.67 d (7)
2_ax_	27.1	1.89 dddd (14, 13, 12, 3)	2	83.5	4.02 dd (7, 9)
2_eq_		2.08 brdd (14, 4)	3	76.0	4.31 dd (9, 9)
3	88.8	3.13 dd (12, 4)	4	76.1	5.14 ddd (9, 9, 5)
4	40.1		5_ax_	64.6	3.72 dd (12, 9)
5	52.8	0.99 dd (12, 2)	5_ec_		4.77 dd (12, 5)
6_ax_	21.3	1.53 dddd (14, 13, 12, 3)			
6_ec_		1.72 brd (14)	1′	105.5	5.08 d (8)
7_ax_	28.4	1.48 dddd (13, 13, 13, 3)	2′	76.5	3.99 dd (8, 9)
7_eq_		1.75 o	3′	76.1	4.08 dd (9, 9)
8	41.0	3.34 brdd (13, 5)	4′	87.5	3.64 dd (9, 9)
9	154.2		5′	71.8	3.75 dq (9, 6)
10	39.8		6′	18.3	1.72 d (6)
11	115.7	5.58 dd (6, 2)			
12_ax_	71.5		1″	105.2	4.95 d (8)
12_ec_		4.97 dd (6, 1)	2″	73.9	4.03 dd (8, 9)
13	58.6		3″	88.2	4.21 dd (9, 9)
14	46.5		4″	70.0	4.02 o
15_a_	36.8	1.40 ddd (12, 12, 9)	5″	78.1	3.99 o
15_b_		1.83 dd (12, 9)	6a″	62.4	4.48 dd (12, 2)
16_a_	36.0	2.66 dd (14, 9)	6b″		4.17 dd (12, 6)
16_b_		2.34 ddd (14, 12, 9)			
17	89.4		1‴	105.9	5.27 d (8)
18	174.8		2‴	75.2	3.98 dd (8, 9)
19	22.7	1.35 s	3‴	88.2	3.70 dd (9, 9)
20	87.1		4‴	70.7	4.13 dd (9, 9)
21	23.1	1.76 s	5‴	78.5	3.94 ddd (9, 6, 3)
22	39.0	2H, 1.90 t (8)	6a‴	62.3	4.44 dd (12, 3)
23_a_	19.1	1.75 o	6b‴		4.26 dd (12, 5)
23_b_		1.64 m	OCH_3_	60.9	3.84 s
24_a_	41.5	1.86 m			
24_b_		1.81 m			
25	82.1				
26	26.2	1.46 s			
27	26.2	1.48 s			
30	16.8	1.06 s			
31	28.2	1.25 s			
32	20.2	1.66 s			
CH_3_CO	22.4	1.95 s			
CH_3_CO	170.3				
OH-17		7.41 s			

a Assignments were confirmed by ^1^H–^1^H-COSY, 1D and 2D-TOCSY, NOESY, HSQC, HSQC-TOCSY, and HMBC experiments. “o” indicates overlapped with other signals.

Compound **2** possesses the same tetrasaccharide chain as holothurin A and scabraside D, identified as 3-*O*-methyl-β-d-glucopyranosyl-(1→3)-β-d-glucopyranosyl-(1→4)-β-d-quinovopyranosyl-(1→2)-4-*O*-sodiumsulfate-β-d-xylopyranoside. In contrast, saponins **1** and **3**–**6** share the same disaccharide chain as holothurin B (**7**), namely, β-d-quinovopyranosyl-(1→2)-4-*O*-sodiumsulfate-β-d-xylopyranoside. These sugar chains are prevalent within the genus *Holothuria*, being present in approximately 32% and 14% of the saponins identified, respectively.^31^ Furthermore, the aglycones present within the isolated saponins comprise six different structures.

Inornatosides A (**1**) and B (**2**) were isolated as a colorless amorphous powder. Their molecular formulas were established as C_43_H_67_O_18_S^–^Na^+^ and C_56_H_89_O_28_S^–^Na^+^, respectively, based on their [M – Na]^−^ ions observed at *m*/*z* 903.4063 (calculated: 903.4048) and *m*/*z* 1241.5249 (calculated: 1241.5261) in the HR-ESI-MS. The ^1^H and ^13^C NMR spectra of both compounds (Tables 1, 2, and 3) displayed resonances consistent with a holothurin-type saponin. Key indicators included seven methyl group singlets, a trisubstituted alkene at δ_H_ = 5.58; δ_C_ = 154.2, 115.7, a lactone carbonyl group (δ_C_ = 174.8), and a broad doublet (δ_H_ = 4.97; δ_C_ = 71.5) ascribed to a methine proton linked to an oxygenated carbon. These features suggested that the aglycone of **1** and **2** has a holostane triterpenoid skeleton with a 9(11)-en-12α-ol moiety.^31^ The HMBC correlations (Figure 3) further confirmed the hypothesis. Specifically, C-5 (δ_C_ = 52.8) correlated with signals assigned to methyl groups H-19, 30, and 31. The first methyl signal showed HMBC correlation with the non-hydrogenated C-9. Moreover, C-13 correlated with the trisubstituted alkene proton H-11, the methyl proton H-32, and a singlet at 7.41 ppm assigned to a hydroxy proton. Additional HMBC correlations from this singlet to C-16, C-17, and C-20 provided evidence for the hydroxylation of C-17.

**Figure 3:**
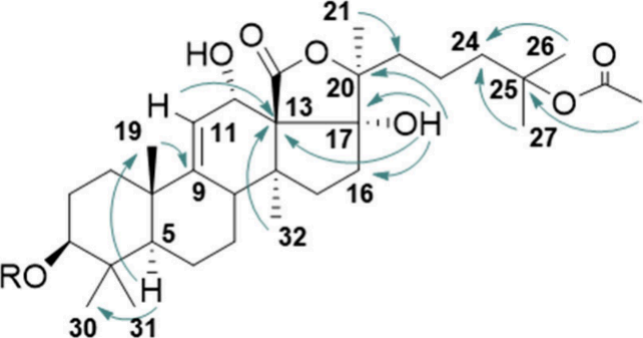
Selected HMBC correlations of inornatoside A (**1**).

A spin system corresponding to a −CH_2_CH_2_CH_2_– moiety was identified from the two-dimensional NMR spectra, connected as a side chain to C-20. HMBC correlations from the methyl group signal H-21 and from two singlets at δ_H_ = 1.46 and 1.48 to carbons within this spin system facilitated the assignment of these latter two singlets as the methyl groups C-26 and C-27. Furthermore, these methyl groups exhibited HMBC correlations with oxygenated carbon C-25 (δ_C_ = 82.1). The ^1^H and ^13^C NMR spectra of compounds **1** and **2** showed characteristic resonances indicative of an acetoxy group (δ_H_ = 1.95 s; δ_C_ = 22.4, 170.3) bonded at C-25. This assignment was strongly supported by a 4-bound HMBC correlation observed between the acetoxy methyl singlet and C-25.

To establish the absolute configuration of the aglycones, it has been assumed that they are holostane-type saponins with a lanostane triterpene skeleton. Specifically, for holothurin B (**7**), the absolute configuration of the holostane skeleton was corroborated by X-ray crystallography by Yuan et al.^32^ The pentacyclic unit (Figure 4) of the aglycone showed correlations in the NOESY experiment. Key cross-peaks observed for protons on the β-face included H-30/H-19 and H-19/H-8. On the α-face, significant correlations were found between H-3/H-5, H-5/H-7ax, H-7ax/H-32, H-32/OH-17, and OH-17/H-21. This latter correlation specifically confirmed the α orientation of the hydroxy group at C-17. Moreover, the NOESY cross-peaks between H-12 and H-21 were consistent with the β orientation of H-12. Thus, it was confirmed that compounds **1** and **2** share the holostane configuration of holothurin B (**7**) identified as 25-acetoxyholost-9(11)-ene-3β,12α,17α-triol.

**Figure 4:**
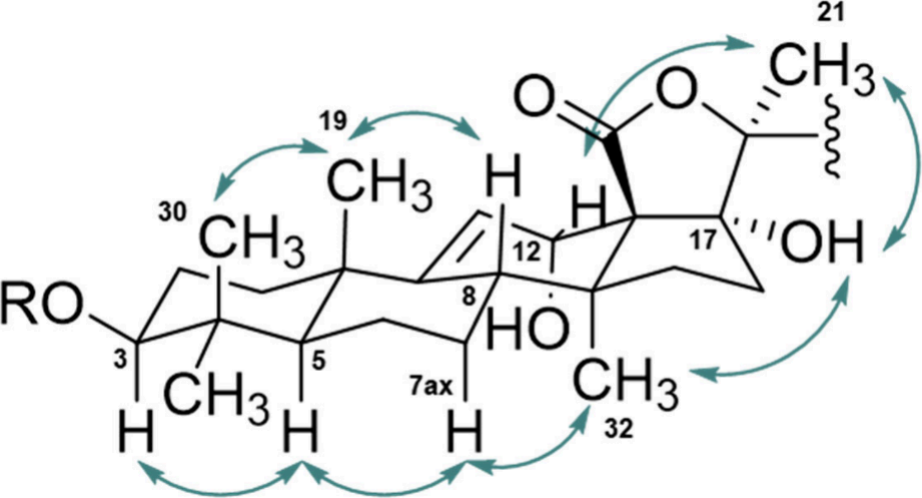
Key NOESY correlations of holothurins **1**–**3** and **6**.

The *O*-glycosidic bond linking the sugar chains at C-3 of the aglycone was confirmed by the observation of an HMBC correlation between the anomeric proton H-1 of xylose and the C-3 of the aglycone. Moreover, the sequence of sugar moieties was further corroborated by analysis of both HMBC and NOESY spectra. Thus, the structure of inornatoside A (**1**) was established to be 25-acetoxy-3-*O*-(β-d-quinovopyranosyl-(1→2)-4-*O*-sodiumsulfate-β-d-xylopyranoside)-holost-9­(11)-ene-3β,12α,17α-triol, and that of inornatoside B (**2**) was found to be 25-acetoxy-3-*O*-(3-*O*-methyl-β-d-glucopyranosyl-(1→3)-β-d-glucopyranosyl-(1→4)-β-d-quinovopyranosyl-(1→2)-4-*O*-sodiumsulfate-β-d-xylopyranoside)-holost-9­(11)-ene-3β,12α,17α-triol.

Inornatoside C (**3**) was obtained as a white, amorphous powder. Its molecular formula was established as C_41_H_65_O_17_S^–^Na^+^ by the [M – Na]^−^ ion at *m*/*z* 861.3941 in the HR-ESI-MS (calcd. 861.3942). The ^1^H and ^13^C NMR spectra (Tables 1 and 2) displayed signals consistent with a holothurin-type saponin possessing a disaccharide sugar chain. A complete assignment of the ^1^H and ^13^C NMR signals of the aglycone moiety of **3** revealed a modification in the side chain as the only structural difference compared to inornatoside A (**1**). Specifically, the signals corresponding to an acetoxy group were absent and the ^1^H NMR signals of methyl groups H-26 and H-27 were significantly shielded. HMBC correlations from these methyl proton signals to δ_C_ = 69.5 supported the presence of a hydroxy group at C-25. The aglycone of **3** was therefore identified as holost-9(11)-ene-3β,12α,17α,25-tetraol. The complete structure of inornatoside C (**3**) was established to be 3-*O*-(β-d-quinovopyranosyl-(1→2)-4-*O*-sodiumsulfate-β-d-xylopyranoside)-holost-9­(11)-ene-3β,12α,17α,25-tetraol.

The ^13^C NMR chemical shift of C-25 in open-chain C-20 holothurins has been a subject of controversy. This debate was highlighted in a recent review,^31^ which provided a thorough analysis of the spectroscopic characteristics of these saponins. Some researchers have reported a C-25 chemical shift of approximately 69 ppm for saponins with a hydroxy group at this position, such as holothurin A_5_,^33^ holothurin B_4_,^30^ marmoratoside B,^34^ and leucospilotaside A.^35^ The signal for C-25 is deshielded, shifting to 81–82 ppm, when this hydroxy group is either acetylated^36^ or replaced by a hydroperoxide group.^37^

Conversely, another group of researchers described saponins, including scabraside D,^25^ hillaside C,^38^ and ananaside B,^39^ as having a hydroxy group at C-25 but with a reported chemical shift of 81 ppm. This is in direct conflict with the first group’s findings. Saponins isolated from *H. inornata* provide crucial evidence to resolve this discrepancy. Two pairs of saponins with aglycones that differed only by the acetylation of the C-25 hydroxy group have been isolated. The acetylated saponins, **1** and **2**, showed a C-25 chemical shift of 82.1 ppm, which is consistent with the deshielded signal expected for an acetyl group. In contrast, deacetylated saponins, **3** and scabraside D, exhibited a C-25 chemical shift of 69.5 ppm. This finding supports the initial reports that a hydroxylated C-25 resonates at approximately 69 ppm and confirms the expected shielding effect upon deacetylation. These findings support the first group of authors, and the chemical shifts of C-25 and other nearby positions in the isolated saponin, which is structurally consistent with the previously described scabraside D, do not match the values reported in the literature.^25^

Inornatoside D (**4**) was obtained as a white, amorphous powder. Its molecular formula was established as C_41_H_61_O_18_S^–^Na^+^ by the [M – Na]^−^ ion at *m*/*z* 873.3575 in the HR-ESI-MS (calcd. 873.3579). Judging by the similarity of the ^1^H and ^13^C NMR spectra, compound **4** exhibited a holostane framework like that of compounds **1** and **3**, including the disaccharide in C-3. A spin system −CH_2_–CH– moiety was identified and assigned to the D ring (Figure 5). The methine group was identified as oxygenated (δ_H_ = 5.01 dd, δ_C_ = 90.3) and was assigned as C-16 due to the HMBC correlation from its proton signal to C-13, C-14, and C-17. Furthermore, the hydroxy proton signal at C-17 (δ_H_ = 7.95 s) correlated with that at C-16. The spin system assigned to the side chain exhibited ^1^H and ^13^C NMR chemical shifts and two-dimensional correlations consistent with a tetrahydrofuran ring like those observed for holothurin B (**7**), with the exception of C-22. A non-hydrogenated carbon signal appearing at δ_C_ = 119.7 correlated with H-21, H-23, and H-24 in the HMBC spectrum, thus leading to its assignment as C-22. The significant downfield shift of this carbon signal suggests that C-22 is doubly oxygenated. To account for the molecular formula of inornatoside D (**4**) and considering the observed signals for the hydroxy protons at C-12 and C-17, a novel cyclic ether linkage between C-22 and C-16 is proposed within the structure, thereby transforming C-22 into a spirostanic position (Figure 5). The NOESY correlations observed for the pentacyclic unit of compound **4** were identical to those of compounds **1**–**3** (Figure 4), consistent with a typical holostane skeleton. Moreover, NOESY correlations between the methyl group protons at C-32 and H-16 confirm the α orientation of H-16. Additionally, the observed correlations between protons of one of the C-25 methyl groups and H-16 support an *S* configuration at C-22 (Figure 5). This newly formed cyclic ether is commonly found in steroidal saponins, specifically spirostanic or furostanic types;^40^ however, it has not been previously described in holothurins. Polyoxygenated steroids with a similar skeletal framework have been isolated from the gorgonian *Isis hippuris*.^41^

**Figure 5:**
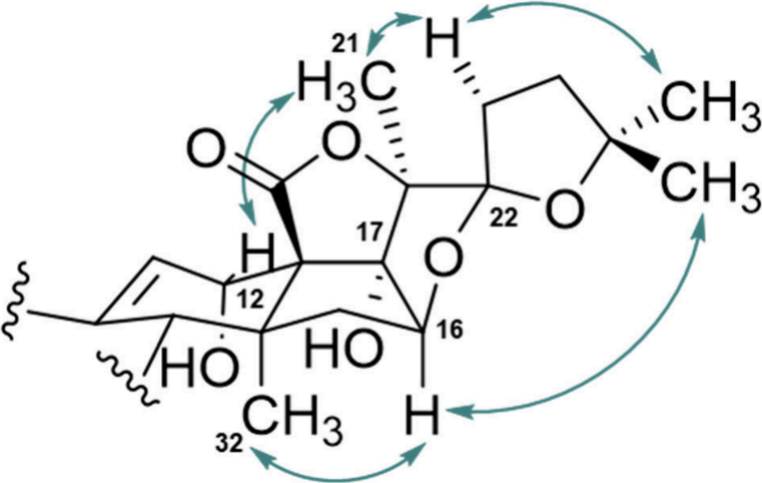
Key NOESY correlations of inornatoside D (**4**).

The structure of inornatoside D (**4**) was established to be 3-*O*-(β-d-quinovopyranosyl-(1→2)-4-*O*-sodiumsulfate-β-d-xylopyranoside)-(16*S*,22*S*)-16,22:22,25-diepoxyholost-9­(11)-ene-3β,12α,17α-triol.

Inornatoside E (**5**) was obtained as a white, amorphous powder. The molecular formula was established as C_36_H_51_O_18_S^–^Na^+^ by the [M – Na]^−^ ion at *m*/*z* = 803.2798 in the HR-ESI-MS (calcd. 803.2796). ^1^H and ^13^C NMR spectra were consistent with a holostane saponin bearing the disaccharide moiety. However, a notable absence of a set of signals characteristic of the aglycone side chain was observed. HMBC correlations were recorded from a carbonyl signal at δ_C_ = 173.6 to H-21 and H-16. Furthermore, the NMR signals for C-16 were found at δ_C_ = 91.8 and δ_H_ = 5.22, establishing the presence of a second γ-lactone ring likely formed between C-16 and C-22. Finally, the observed NOESY correlations of compound **5** are identical to those of compound **4** and consistent with a holostane-type aglycone. The structure of inornatoside E (**5**) was thus established to be 3-*O*-(β-d-quinovopyranosyl-(1→2)-4-*O*-sodiumsulfate-β-d-xylopyranoside)-(16*S*)-3β,12α,17α-trihydroxy-23,24,25,26,27-pentanorholost-9­(11)-ene-22­(16)-lactone.

Saponins featuring shortened side chains are rarely encountered in sea cucumbers.^42^ Among these, those lacking the characteristic 18(20)-lactone are classified as non-holostane glycosides. In the structure of inornatoside E (**5**), this lactone is conserved, and a new one, 22(16)-lactone, is also present.

Compound **6** and holothurin B (**7**) exhibited identical HR-ESI-MS spectra, indicating that they are isomers. A comparison of their ^13^C NMR spectra revealed identical structural features for the sugar chain and the A, B, and C rings. However, the chemical shifts of signals corresponding to positions 16 to 27 in the ^1^H and ^13^C NMR spectra of compound **6** were shifted. The assignment of each signal within this part of the saponin, facilitated by two-dimensional HSQC and HMBC spectra, indicates that compound **6** possesses the same carbon skeleton as holothurin B (**7**). Therefore, compound **6** is hypothesized to possess a difference in configuration at one of three chiral centers: C-17, C-20, or C-22. A singlet at 7.48 ppm was assigned to the hydroxy proton at C-17, based on its correlation with this carbon in the HMBC spectrum. Furthermore, this hydroxy proton signal exhibited a NOE correlation with the C-32 methyl group, indicating its α-orientation on the aglycone, as in holothurin B (**7**). Additionally, a typical NOE between H-12 and H-21 signals, observed in holothurins, was found, suggesting that the lactone ring retains the same spatial arrangement at C-20. Finally, it is proposed that compound **6** is the C-22 epimer of holothurin B (**7**).

Conformational analysis (Figure 6) revealed that for both compounds H-22 adopts a spatial position in close proximity to the H-16,^32^ potentially minimizing steric repulsions in this region. This observation is corroborated by the NOE correlation observed between H-16 and H-22 in both compounds. Finally, the NOE correlation observed between H-27 and H-16 confirms the *S* configuration at C-22 for holothurin B (**7**), whereas the NOE correlation observed between H-23 and H-16 indicates an *R* configuration at C-22 for compound **6**. Based on these findings, compound **6** is determined to be the C-22 epimer of holothurin B (**7**) and is therefore designated as (22*R*)-holothurin B (**6**), representing, to our knowledge, the first reported instance of such an epimer.

**Figure 6:**
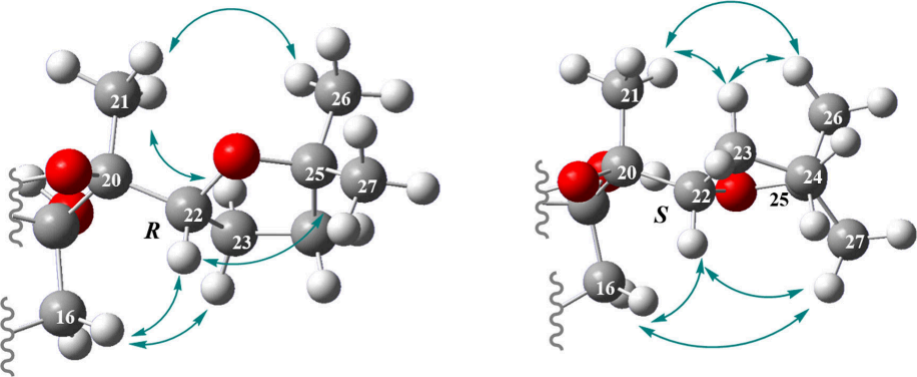
Key NOESY correlations for 22*R* and 22*S* epimers of holothurin B (**6**, **7**) on the lowest energy conformer representations. The structures are truncated to show the area of interest.

### Cytotoxic Activity of the Isolated Compounds

The cytotoxic activity of the extract of saponins, the enriched fraction of saponin, and the major compounds **1**, **2**, and **7** was determined at a concentration of 25 μg/mL (Figure 1) based on their growth inhibition effect on the cancer cell lines human glioblastoma (U251), human prostatic adenocarcinoma (PC-3), human chronic myelogenous leukemia (K562), human colorectal adenocarcinoma (HCT-15), human mammary adenocarcinoma (MCF-7), and human lung adenocarcinoma (SKLU-1) using the SRB method. The results indicate that the enriched fraction of saponins exhibits greater antiproliferative activity than the saponin extract, while for the pure compounds, compounds **1** and **2** are significantly more active than holothurin B (**7**). Except for the K562 cell line, overall, the cell lines used in this study did not exhibit high sensitivity to isolated holothurin B (**7**). This saponin (**7**) is a well-characterized triterpene glycoside^26^ known for its cytotoxic activity against various cancer cell lines. Studies have demonstrated the effectiveness of holothurin B (**7**) from *H. edulis* against human prostate cancer (LNCaP), liver cancer (HepG2), oral epidermoid carcinoma (KB), breast cancer (MCG-7), and melanoma (SK-Mel2) cell lines with IC_50_ values between 2.66 and 3.66 μM;^37^ moreover, holothurin B (**7**) from *H. moebii* inhibits the proliferation of colorectal cancer cells (HCT-HCT-8, HCT-15, HCT-116, and SW620) with IC_50_ values between 2.14 and 2.92 μM.^43^ Holothurin B (**7**) from *H. scabra* inhibits cervical cancer (HeLa), hepatoma (HepG2), and leukemia (K562) cell lines with IC_50_ values between 1.79 and 3.64 μg/mL.^44^ Considering the growth inhibition observed at 25 μg/mL (Figure 1), compounds **1** and **2** were chosen for the IC_50_ determination (Table 4).

**Table 4:** Cytotoxic Activity of Compounds **1** and **2**

	Cell Line IC_50_ (μM ± SD^a^)					
Compound	U251	PC-3	K562	HCT-15	MCF-7	SKLU-1
Inornatoside A (**1**)	3.53 ± 0.13	5.35 ± 0.01	4.24 ± 0.02	4.69 ± 0.01	>50.0	3.65 ± 0.02
Inornatoside B (**2**)	0.54 ± 0.01	0.49 ± 0.01	0.48 ± 0.01	1.04 ± 0.01	0.47 ± 0.04	0.50 ± 0.01
Mitoxantrone^b^	0.55 ± 0.04	0.63 ± 0.04	0.65 ± 0.02	0.75 ± 0.04	0.72 ± 0.01	1.16 ± 0.15

a Standard deviation.

b Positive control.

Inornatoside A (**1**) exhibited moderate activity, with IC_50_ values between 3.53 and 5.35 μM, compared to mitoxantrone, which showed IC_50_ values between 0.55 and 1.16 μM. Meanwhile, inornatoside B (**2**) exhibited stronger cytotoxic activity, with IC_50_ values ranging from 0.47 and 1.04 μM, with the highest activity observed against the MCF-7 cell line (0.47 μM), compared to mitoxantrone.

Several studies have investigated the structure–activity relationship of sea cucumber saponins. It has been demonstrated that saponins containing a combination of linear tetrasaccharide chains and a holostane-type aglycone exhibit enhanced anticancer activity.^45^ Additionally, the presence and position of sulfate groups in the sugar chain can further enhance the bioactivity of these compounds.^28^ This trend is evident in the sulfated tetrasaccharide inornatoside B (**2**), which displays greater cytotoxic activity in the evaluated cancer cell lines, with IC_50_ values ranging from 0.47 to 1.04 μM. In contrast, the sulfated disaccharide inornatoside A (**1**), which shares the same holostane-type aglycone, exhibits lower activity (Table 4).

In summary, nine sulfated saponins were isolated from the body wall of *Holothuria inornata*. Six of these compounds represent new holothurins, named inornatosides A–E (**1**–**5**) and 22*R*-holothurin B (**6**). Notably, inornatosides D (**4**) and E (**5**) feature rare aglycones incorporating a new 22(16)-ring. The remaining saponins, scabraside D, holothurin B (**7**), and holothurin A, have been previously reported in other *Holothuria* species.^31^ Various ecological factors can influence the biosynthesis of saponins in sea cucumbers, contributing to variability in the composition of these glycosides. This chemical diversity has proven useful for taxonomic classification within the *Holothuria* genus. Inornatosides A (**1**) and B (**2**) identified in *H. inornata* can serve as chemotaxonomic markers, acting as fingerprints for the precise identification of this species. Additionally, inornatoside B (**2**) demonstrated significant growth-inhibitory activity against all tested cancer cell lines, with IC_50_ values of approximately 0.50 μM in most cases. Moreover, its IC_50_ values for human mammary adenocarcinoma (MCF-7) and human lung adenocarcinoma (SKLU-1) cell lines surpassed those of the positive control.

## Experimental Section

### General Experimental Procedures

Optical rotations were measured on a JASCO P-2000 digital polarimeter (Maryland, USA) using methanol or water as the solvent. The IR spectra were collected on a Thermo Fisher FTIR-NICOLET IS-50 spectrometer (California, USA). The exact masses were measured on a UPLC-QTOF ESI (Waters Xevo G2, Manchester, UK) high-resolution mass spectrometer (HRESI-TOFMS), and a Waters Acquity UPLC HSS T3 column (1.8 μm, 2.1 mm × 150 mm) was used. The 1D and 2D NMR spectra were recorded on a Bruker AVANCE NEO (Rheinstetten, Germany) at 700 MHz with a 5 mm helium-cooled cryoprobe. The ^1^H and ^13^C NMR spectra were recorded on samples dissolved in pyridine-*d*_5_ (Eurisotop, Saint Aubin, France). The chemical shifts are given on the δ scale and are referred to the residual pyridine signals (δ_H_ 8.70, 7.55, 7.18 and δ_C_ 149.84, 135.60, 123.48).

HPLC grade organic solvents such as *n*-hexane and acetone were purchased from VWR chemicals (Leuven, Belgium). Acetonitrile (ACN), methanol (MeOH), and dichloromethane (CH_2_Cl_2_) were purchased from Fischer Scientific (Madrid, Spain), and acetic acid and *n*-butanol were purchased from PanReac (Darmstadt, Germany). Ammonium acetate was purchased from Merck (Darmstadt, Germany). Trifluoroacetic acid was purchased from Fisher Scientific (Madrid, Spain). Ultrapure water from a Milli-Q system (Millipore, Bedford, MA, USA) was used to prepare all aqueous solutions. Thin-layer chromatography (TLC, Silica gel F_254_S, RP-18) and preparative TLC (PTLC, Silica gel F_254_S, RP-18, 0.25 mm) chromatoplates were purchased from Merck (Darmstadt, Germany). The compounds were detected by spraying the chromatoplate with H_2_SO_4_/H_2_O/HOAc (4:16:80). Amberlite XAD-4 adsorbent resin from Merck (Darmstadt, Germany) was used in the solid-phase extraction for sample desalination. LiChroprep RP-18 (40–63 μm) from Merck (Darmstadt, Germany) was used for vacuum column chromatography for the first fractionation. HPLC purifications were performed using a VWR Hitachi Chromaster (VWR International, Pennsylvania, USA), equipped with a HITACHI VWR 5160 binary pump, a HITACHI VWR 5310 temperature-controlled column compartment, and a HITACHI VWR 5430 UV-DAD detector; the detection wavelength was set at 210 nm. The control and data acquisition system were carried out with Chromaster software, version 2.0. An analytical LiChrospher 100 RP-8 end-capped (5 μm) column (4 mm × 250 mm) was used. The flow rate was set at 1.0 mL/min.

### Sea Cucumber Material

The specimens were collected in Mazatlán, located in Sinaloa, Mexico (coordinates: 23° 18′ 30.28″ N, 106° 29′ 28.63″ W) on June 21st, 2022. The collection was carried out by autonomous diving at depths of 1 to 5 m. The species was identified at the Marine Sciences and Limnology Institute with the support of the Echinoderm Molecular Systematics Laboratory based on spicule analysis present on the body wall, for which a sample was taken from the back of the organism, placed on a slide adding 3 drops of a 7% sodium hypochlorite solution (the solution was left for 1 min), and observed with a microscope (40× magnification) after removing the solution; the morphology of the spicules was compared with previously reported data for this species in the literature.^46^ The sea cucumbers identified as *H. inornata* were frozen with liquid nitrogen, transported to the laboratory, and stored in an ultrafreezer (−50 °C) before analysis. A sample was deposited in the National Echinoderm Collection (Mexico) with voucher number ICML-UNAM 18500.

### Extraction and Isolation

The body wall of a sea cucumber was separated from the viscera. Subsequently, the body wall (350 g fresh weight) was ground and mixed with 1.5 L of MeOH/H_2_O in a 7:3 ratio for the extraction of glycosides, followed by stirring for 24 h at 4 °C. The resulting extract was centrifuged at 20,000 rpm and dried using a rotary evaporator. This extraction procedure was repeated three times, yielding a crude extract with a dry weight of 21.8 g. Liquid–liquid extraction was then performed to separate the less polar compounds using *n*-hexane/MeOH/H_2_O followed by CH_2_Cl_2_/MeOH/H_2_O in ratios of 10:9:1 and 5:4:1, respectively. Finally, the aqueous extract was concentrated using a rotary evaporator to yield 15.2 g. Subsequently, this extract was desalted using the polymeric adsorbent Amberlite XAD-4 (300 g) in a chromatographic column (7.0 cm × 40 cm). For salt removal, the extract was washed with 100% deionized H_2_O. The desalination process was confirmed by adding 0.5 mL of 10% AgNO_3_ to 2 mL of the eluate, which did not produce any precipitate. The glycoside-enriched fraction was then recovered by adding 100% MeOH (2 L) and concentrated using a rotary evaporator to yield 1.2 g (0.34%).

This glycoside-enriched fraction was further purified by vacuum column chromatography (6.8 cm × 6.0 cm) with LiChroprep RP-18 (116.1 g), which was conditioned by adding 300 mL of MeOH followed by 300 mL of water. The enriched extract was loaded onto the column and dissolved in 5 mL of water and then eluted with 1 L of 100% Milli-Q water. Subsequently, 50 mL eluates were collected by applying a gradient elution with H_2_O/acetone in the following ratios: 85:15 (eluates 1–12), 80:20 (eluates 13–24), 75:25 (eluates 25–170), 70:30 (eluates 171–243), 65:35 (eluates 244–292), 40:60 (eluate 293), and finally, 1 L of MeOH was added (eluate 294). The obtained fractions were A (39–43, 13.9 mg), B (44–55, 21.7 mg), C (56–77, 30.6 mg), D (78–95, 21.8 mg), E (96–152, 34.8 mg), F (153–212, 166.7 mg), G (213–291, 134.8 mg), and H (292–294, 180.4 mg). Fraction H was composed of inornatoside A (**1**). Fraction G is a 1:1 mixture of inornatoside A (**1**) and holothurin B (**7**). 24.5 mg of fraction F was chromatographed by preparative TLC on reverse-phase silica gel plates (RP-18, F254s), using H_2_O/acetone (1:1) as the mobile phase, yielding 10.2 mg of inornatoside B (**2**) and 10.8 mg of holothurin B (**7**).

Fraction A was separated by HPLC with H_2_O/MeOH/AcONH_3_ (1 M aqueous solution) in a ratio of 45:54:1 as the mobile phase, obtaining the compound scabraside D (0.7 mg, t_R_ 13.3 min). In the case of fraction B, the same solvent with a ratio 41:58:1, was used as the mobile phase, resulting in subfraction B1 (3.1 mg, t_R_ 8.1 min) and 1.5 mg of holothurin A. Further purification of B1 using a ratio of 45:54:1 in the mobile phase led to the isolation of inornatoside E (**5**) (0.4 mg, t_R_ 12.1 min). HPLC of fraction C using a ratio 41:58:1 in the mobile phase led to holothurin A (4.8 mg, t_R_ 15.8 min) and inornatoside C (**3**) (6.6 mg, t_R_ 17.7 min). With same HPLC conditions, 14.9 mg of fraction D was analyzed yielded 4.1 mg of inornatoside C (**3**) (t_R_ 16.7 min), 1.1 mg of inornatoside D (**4**) (t_R_ 24.3 min), and 1.4 mg of inornatoside B (**2**) (t_R_ 27.2 min). 16.0 mg of fraction E was injected into HPLC using H_2_O/MeOH/AcONH_3_ (1 M aqueous solution) (39:60:1) as mobile phase to obtain inornatoside B (**2**) (5.3 mg, t_R_ 13.3 min), 22*R*-holothurin B (**6**) (0.5 mg, t_R_ 18.1 min), and holothurin B (**7**) (1.3 mg, t_R_ 27.3 min).

### Acid Hydrolysis of Fraction F

Fraction F (37 mg) was heated in 20 mL of 2 M trifluoroacetic acid (100 °C, 1 h) and dried under vacuum. The crude was suspended in H_2_O/EtOAc and then extracted with EtOAc. The aqueous layer, which contains sugars, was dried under a vacuum to obtain 14 mg. The sample was purified by preparative Si gel TLC (CHCl_3_/MeOH/H_2_O, 50:20:2) to afford quinovose [2.2 mg, *R*_*f*_ = 0.48, [α]^26^_D_ +25.9 (c 0.18, H_2_O)]; xylose [2.5 mg, *R*_*f*_ = 0.35, [α]^26^_D_ +19 (c 0.21, H_2_O)]; and glucose [1.3 mg, Rf = 0.20, [α]^26^_D_ +53 (c 0.11, H_2_O)], which were identified by comparison with authentic samples.

#### Inornatoside A (**1**)

[α]^25^_D_ −6.4 (*c* 0.14, MeOH); IR ν_max_ 3306, 2945, 2872, 1762, 1732, 1646, 1540, 1451, 1369, 1249, 1220, 1049, 1006, 980 cm^–1^; ^1^H and ^13^C NMR, see Tables 1 and 2; HRESIMS (negative ion mode) *m*/*z* 903.4063 [M – Na]^−^ (calcd for C_43_H_67_O_18_S^–^, 903.4048). MS^*n*^ *m*/*z* 843 [M – Na – 60]^−^, 825 [M – Na – 60 – 18]^−^.

#### Inornatoside B (**2**)

[α]^25^_D_ −5.9 (c 0.37, MeOH); IR ν_max_ 3347, 2967, 2945, 2867, 1737, 1441, 1362, 1214, 1115, 1048, 1008, 968 cm^–1^; ^1^H and ^13^C NMR, see Table 3; HRESIMS (negative ion mode), *m*/*z* 1241.5249 [M – Na]^−^ (calcd for C_56_H_89_O_28_S^–^, 1241.5261). MS^*n*^ *m*/*z* 1181 [M – Na – 60]^−^, 1163 [M – Na – 60 – 18]^−^.

#### Inornatoside C (**3**)

[α]^25^_D_ −7.3 (c 0.04, MeOH); IR ν_max_ 3318, 2938, 2869, 1729, 1453, 1363, 1214, 1054, 1009, 967 cm^–1^; ^1^H and ^13^C NMR, see Tables 1 and 2; HRESIMS (negative ion mode), *m*/*z* 861.3937 [M – Na]^−^ (calcd for C_41_H_65_O_17_S^–^, 861.3942). MS^*n*^ *m*/*z* 843 [M – Na – 18]^−^.

#### Inornatoside D (**4**)

[α]^25^_D_ −13.7 (c 0.09, MeOH); ^1^H and ^13^C NMR, see Tables 1 and 2; HRESIMS (negative ion mode), *m*/*z* 873.3575 [M – Na]^−^ (calcd for C_41_H_61_O_18_S^–^, 873.3579). MS^*n*^ *m*/*z* 855 [M – Na – 18]^−^.

#### Inornatoside E (**5**)

[α]^25^_D_ −5.8 (c 0.03, MeOH); ^1^H and ^13^C NMR, see Tables 1 and 2; HRESIMS (negative ion mode), *m*/*z* 803.2798 [M – Na]^−^ (calcd for C_36_H_51_O_18_S^–^, 803.2796). MS^*n*^ *m*/*z* 785 [M – Na – 18]^−^, 741 [M – Na – 18 – 44]^−^.

#### 22*R*-Holothurin B (**6**)

[α]^25^_D_ −13.2 (c 0.04, MeOH); ^1^H and ^13^C NMR, see Tables 1 and 2; HRESIMS (negative ion mode), *m*/*z* 859.3777 [M – Na]^−^ (calcd for C_41_H_63_O_17_S^–^, 859.3786). MS^*n*^ *m*/*z* 841 [M – Na – 18]^−^.

### UPLC-QTOF/MS^*n*^ Analysis

100 ppm of the saponins was prepared in H_2_O/MeOH (1:1). Before injection, the sample was filtered through a PTFE syringe filter (0.22 μm). The injection volume was 5 μL. A Waters Acquity UPLC HSS T3 column (1.8 μm, 2.1 mm × 150 mm) and VanGuard precolumn were used and maintained at a temperature of 45 °C. The mobile phase consisted of H_2_O (A) and CH_3_CN (B), each containing 0.1% (v/v) formic acid; gradient of the mobile phase used is as follows: 0–0.5 min, 95–70% A; 0.5–6.0 min, 70–50% A; 6.0–7.0 min, 50–5% A; 7.0–7.5 min, 5% A; 7.5–8.0 min, 5–95% A; 8.0–10–0 min, 95% A. Eluent flow rate was 0.4 mL/min. The temperature in the autosampler was set at 10 °C.

The mass analysis by electrospray ionization was obtained in negative ion mode (ESI-). The equipment conditions were as follows: capillary voltage 2800 V, sampling cone voltage 40 V, source temperature 120 °C, desolvation temperature 500 °C, Desolvation gas flow 850 L/h, and cone gas flow 20 L/h. The mass spectra were acquired in centroid mode using MS^*n*^ (collision energy, 6 eV, and collision energy ramp, 90–150 eV) over a mass range 100–2000 *m*/*z*, retention time range 0–10 min with a scan time of 0.5 s. The data were acquired and processed using MassLynx version 4.1 (Waters Inc. Milford, MA, USA). Retention times of saponins: **1**: 7.84 min, **2**: 6.06 min, **3**: 5.27 min, **4**: 4.44 min, **5**: 6.97 min, **6**: 7.23 min, **7**: 7.61 min, scabraside D: 3.71 min, holothurin A: 5.54 min.

### Cytotoxicity Assay

Cytotoxicity was evaluated based on the percentage of cell growth inhibition. The cancer cell lines were obtained from the National Cancer Institute (NCI, Bethesda, MD). The study utilized human cancer cell lines derived from central nervous system glia (U251), prostate (PC-3), leukemia (K562), colon (HCT-15), breast (MCF-7), and lung (SKLU). Each cell line was maintained in RPMI 1640 medium supplemented with fetal bovine serum (10%), l-glutamate (2.0 mM), 10,000 units/mL penicillin G, 10,000 mg/mL streptomycin sulfate, 25 mg/mL amphotericin B and 1% nonessential amino acids. Cytotoxicity was assessed by treating cancerous cells with the extract, the saponin fraction, and the major isolated compounds, all of which were solubilized in ultrapure water (18.2 MΩ) and tested at a concentration of 25 μg/mL. For the determination of IC_50_ values, the same cancer cell lines were evaluated and had the same treatment, while the range of concentrations was 25, 12.5, 6.5, 5.0, 2.5, and 1.25 μg/mL in triplicate wells for each concentration; commercially formulated mitoxantrone, used as a water-soluble chemotherapeutic drug, was used as a positive control, and ultrapure water was used as a negative control. The resulting data obtained from the assay for each cell line were analyzed using nonlinear regression to calculate IC_50_ using GraphPad Prism 10.5.0 (San Diego, CA, USA). The percentage of cell growth inhibition was determined by using the protein-binding dye sulforhodamine B (SRB) in a microculture assay. Cells were harvested from culture flasks and seeded into 96-well plates at a density of 5,000 to 10,000 cells per well, maintaining the same temperature and humidity conditions as during culture incubation. The test compounds were then added, and cells were exposed for 48 h. After incubation, cells were fixed in situ by adding 50 μL of cold 50% trichloroacetic acid and incubating at 4 °C for 1 h. The plates were then washed with Milli-Q water, air-dried at room temperature, and stained with a 0.4% SRB solution. Excess SRB was removed by washing with 1% aqueous acetic acid. The plates were air-dried, and the bound dye was solubilized with 100 μL of a 10 mM unbuffered Tris base. The plates were then shaken for 10 min, and absorbance was measured at 515 nm using a microplate spectrophotometer.

## Supplementary Material



## Data Availability

The NMR data for compounds **1**–**6** has been deposited in the Natural Products Magnetic Resonance Database (NP-MRD; www.np-mrd.org) and can be found as NP0351123 (**1**, inornatoside A), NP0351128 (**2**, inornatoside B) NP0351124 (**3**, inornatoside C), NP0351125 (**4**, inornatoside D), NP0351126 (**5**, inornatoside E), and NP0351127 (**6**, 22*R*-holothurin B).

## Notes

HRESIMS and 1D and 2D NMR spectra for compounds **1**–**6**, Comparison of ^1^H NMR signals for disaccharides and tetrasaccharides of saponins, and ^13^C and ^1^H NMR data of known compounds (PDF)
