# Supplementary material for: Cytotoxic Triterpene Glycosides from Mexican Sea Cucumber Holothuria inornata
Source: J Nat Prod. 2025 Sep 22;88(10):2372–82. doi: 10.1021/acs.jnatprod.5c00716 (PMC12560079; doi:10.1021/acs.jnatprod.5c00716)
Supplement: Supplementary file 1 [file np5c00716_si_001.pdf]

Supporting information

CYTOTOXIC TRITERPENE GLYCOSIDES FROM MEXICAN SEA CUCUMBER *HOLOTHURIA INORNATA*.

Esteban López-Sampedro [1,2,3], Roberto Arreguin-Espinosa[3*], Ana M. Simonet[2*]

[1]Posgrado en Ciencias del Mar y Limnología, Universidad Nacional Autónoma de México; Av. Universidad 3000, Ciudad Universitaria Coyoacán, C.P. 04510, Ciudad de México, México

[2]Department of Organic Chemistry, Institute of Biomolecules (INBIO), Campus de Excelencia Internacional (ceiA3), School of Science, University of Cadiz, 11510 Puerto Real, Cadiz, Spain.

[3] Departamento de Química de Biomacromoléculas, Instituto de Química, Universidad Nacional Autónoma de México, Av. Universidad 3000, Circuito Exterior s/n, Coyoacán, Ciudad Universitaria, México City 04510, México

Ana M. Simonet. ana.simonet@uca.es

Roberto Arreguin-Espinosa. arrespin@unam.mx

Table of Contents

Figure S1. IR and HRESI MS[E] (negative mode) of Inornatoside A (1) 3

Figure S2. ^{1}H NMR spectrum of Inornatoside A (1) (700 MHz, Pyridine-*d5*) 4

Figure S3. ^{13}C NMR spectrum of Inornatoside A (1) (700 MHz, Pyridine-*d5*) 5

Figure S4. HSQC spectrum of Inornatoside A (1) (700 MHz, Pyridine-*d5*) 6

Figure S5. HMBC spectrum of Inornatoside A (1) (700 MHz, Pyridine-*d5*) 7

Figure S6. IR and HRESI MS[E] (negative mode) of Inornatoside B (2) 8

Figure S7. ^{1}H NMR spectrum of Inornatoside B (2) (700 MHz, Pyridine-*d5*) 9

Figure S8. ^{13}C NMR spectrum of Inornatoside B (2) (700 MHz, Pyridine-*d5*) 10

Figure S9. HSQC spectrum of Inornatoside B (2) (700 MHz, Pyridine-*d5*) 11

Figure S10. HMBC spectrum of Inornatoside B (2) (700 MHz, Pyridine-*d5*) 12

Figure S11. IR and HRESI MS[E] (negative mode) of Inornatoside C (3) 13

Figure S12. ^{1}H NMR spectrum of Inornatoside C (3) (700 MHz, Pyridine-*d5*) 14

Figure S13. ^{13}C NMR spectrum of Inornatoside C (3) (700 MHz, Pyridine-*d5*) 15

Figure S14. HSQC spectrum of Inornatoside C (3) (700 MHz, Pyridine-*d5*) 16

Figure S15. HMBC spectrum of Inornatoside C (3) (700 MHz, Pyridine-*d5*) 17

Figure S16. HRESI MS[E] (negative mode) of Inornatoside D (4) 18

Figure S17. ^{1}H NMR spectrum of Inornatoside D (4) (700 MHz, Pyridine-*d5*) 19

Figure S18. ^{13}C NMR spectrum of Inornatoside D (4) (700 MHz, Pyridine-*d5*) 20

Figure S19. HSQC spectrum of Inornatoside D (4) (700 MHz, Pyridine-*d5*) 21

Figure S20. HMBC spectrum of Inornatoside D (4) (700 MHz, Pyridine-*d5*) 22

Figure S21. HRESI MS[E] (negative mode) of Inornatoside E (5) 23

Figure S22. ^{1}H NMR spectrum of Inornatoside E (5) (700 MHz, Pyridine-*d5*) 24

Figure S23. ^{13}C NMR spectrum of Inornatoside E (5) (700 MHz, Pyridine-*d5*) 25

Figure S24. HSQC spectrum of Inornatoside E (5) (700 MHz, Pyridine-*d5*) 26

Figure S25. HMBC spectrum of Inornatoside E (5) (700 MHz, Pyridine-*d5*) 27

Figure S26. HRESI MS[E] (negative mode) of (22R)-Holothurin B (6) 28

Figure S27. ^{1}H NMR spectrum of (22R)-Holothurin B (6) (700 MHz, Pyridine-*d5*) 29

Figure S28. ^{13}C NMR spectrum of (22R)-Holothurin B (6) (700 MHz, Pyridine-*d5*) 30

Figure S29. HSQC spectrum of (22R)-Holothurin B (6) (700 MHz, Pyridine-*d5*) 31

Figure S30. HMBC spectrum of (22R)-Holothurin B (6) (700 MHz, Pyridine-*d5*) 32

Figure S31. ^{1}H NMR spectra comparison: Signals of disaccharides of compounds 1, 3-6 and 7 (700 MHz, Pyridine-*d5*) 33

Figure S32. Figure S32. ^{1}H NMR spectra comparison: Signals of tetrasaccharides of compounds 2, scabraside D and holothurin A (700 MHz, Pyridine-*d5*). 34

Table S1. ^{13}C and ^{1}H NMR data (*J* in Hz) for the aglycone moieties of compounds holothurins A and B and scabroside D (Pyridine-*d5*) 35

Table S2. ^{13}C and ^{1}H NMR data (*J* in Hz) for the sugar chain moieties of compounds holothurins A and B and scabroside D (Pyridine-*d5*) 36

Elemental Composition Report **Page 1**

Tolerance = 3.0 mDa / DBE: min = -1.5, max = 50.0
Element prediction: Off
Number of isotope peaks used for i-FIT = 3

Monoisotopic Mass, Even Electron Ions
658 formula(e) evaluated with 4 results within limits (up to 50 closest results for each mass)
Elements Used:
C: 1-500 H: 1-1000 O: 1-100 S: 0-1
HI-PM1-A1-2-NEG 440 (7.871)
1: TOF MS ES-

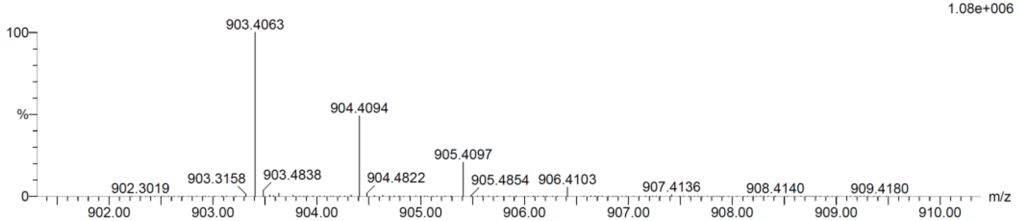

	RA	Calc. Mass	mDa	PPM	DBE	i-FIT	Norm	Conf(%)	Formula
Minimum:	80.00				-1.5				
Maximum:	100.00		3.0	10.0	50.0				
Mass	RA	Calc. Mass	mDa	PPM	DBE	i-FIT	Norm	Conf(%)	Formula
903.4063	100.00	903.4048	1.5	1.7	10.5	613.8	0.000	100.00	C43 H67 O18 S

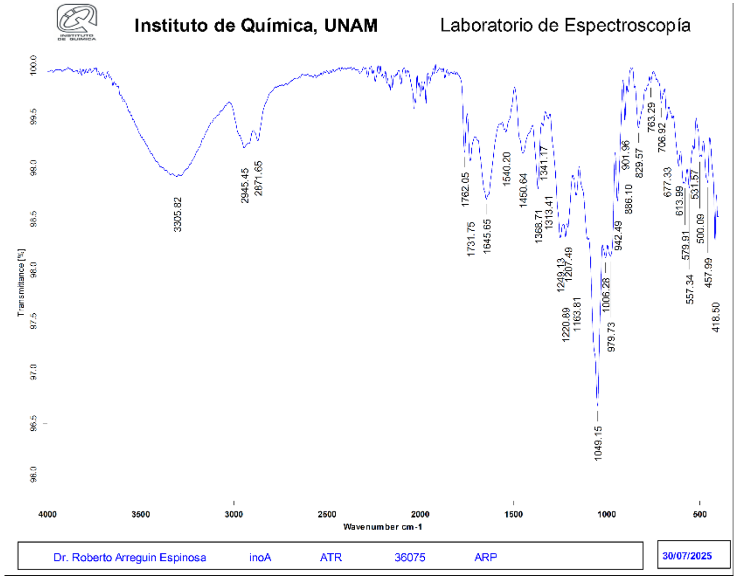

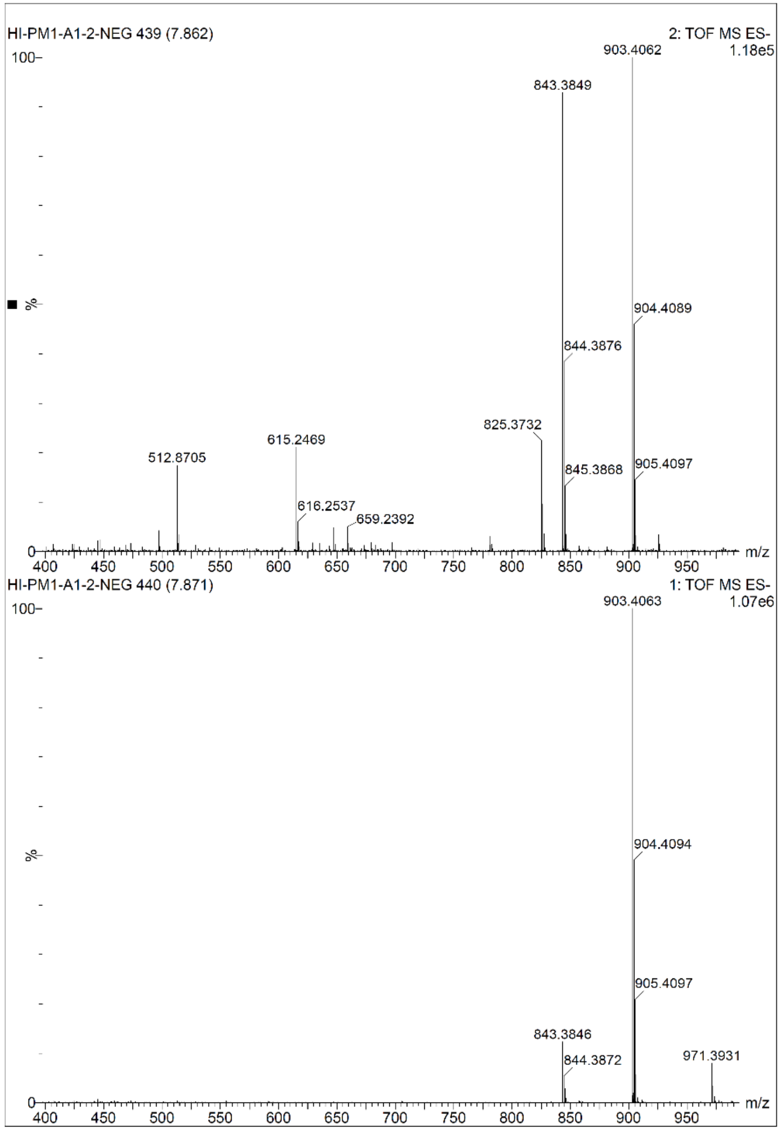

Figure S1. IR and HRESI MS^E (negative mode) of Inornatoside A (1).

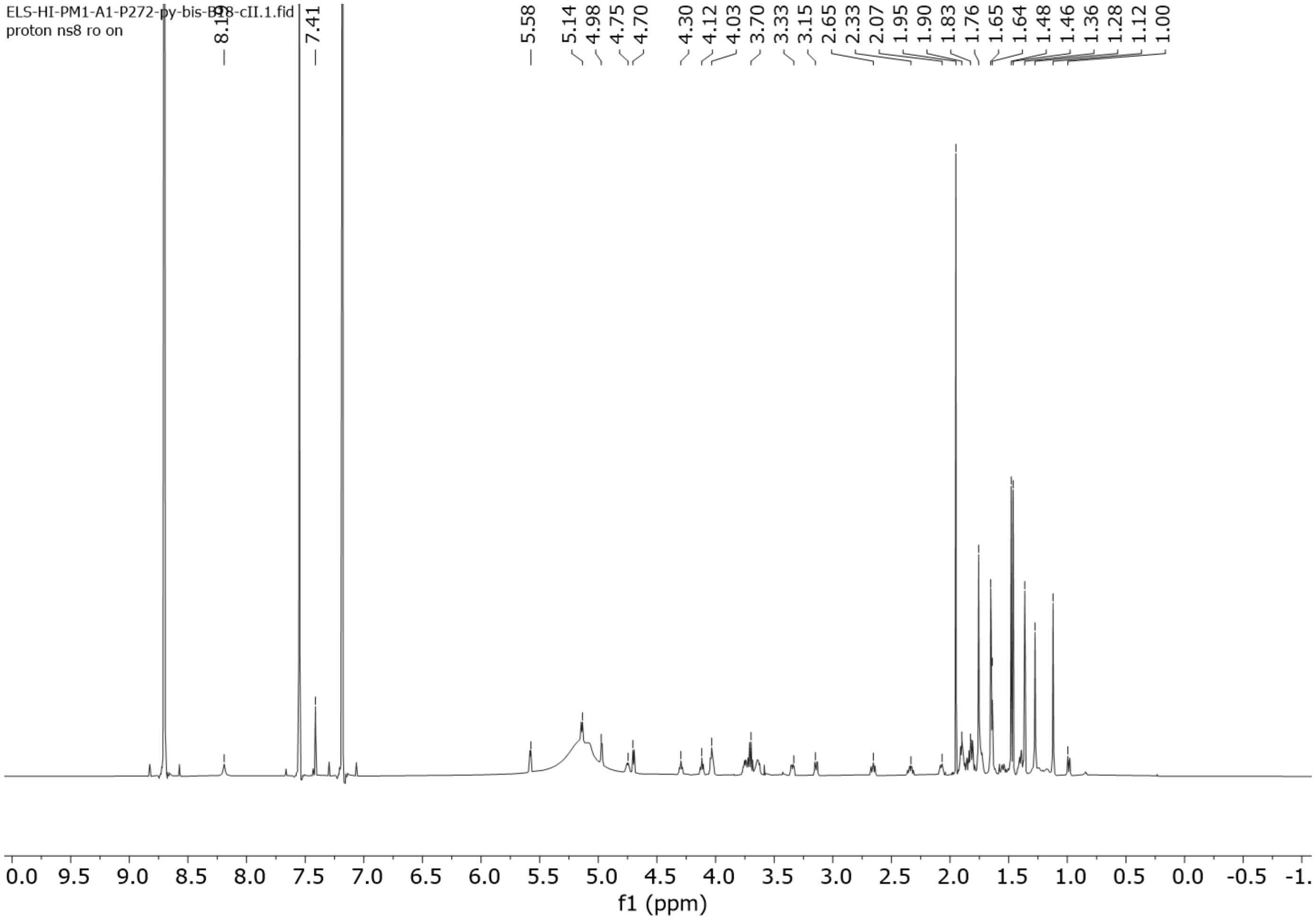

Figure S2. **^{1}H NMR spectrum of Inornatoside A (1) (700 MHz, Pyridine-d_5)**

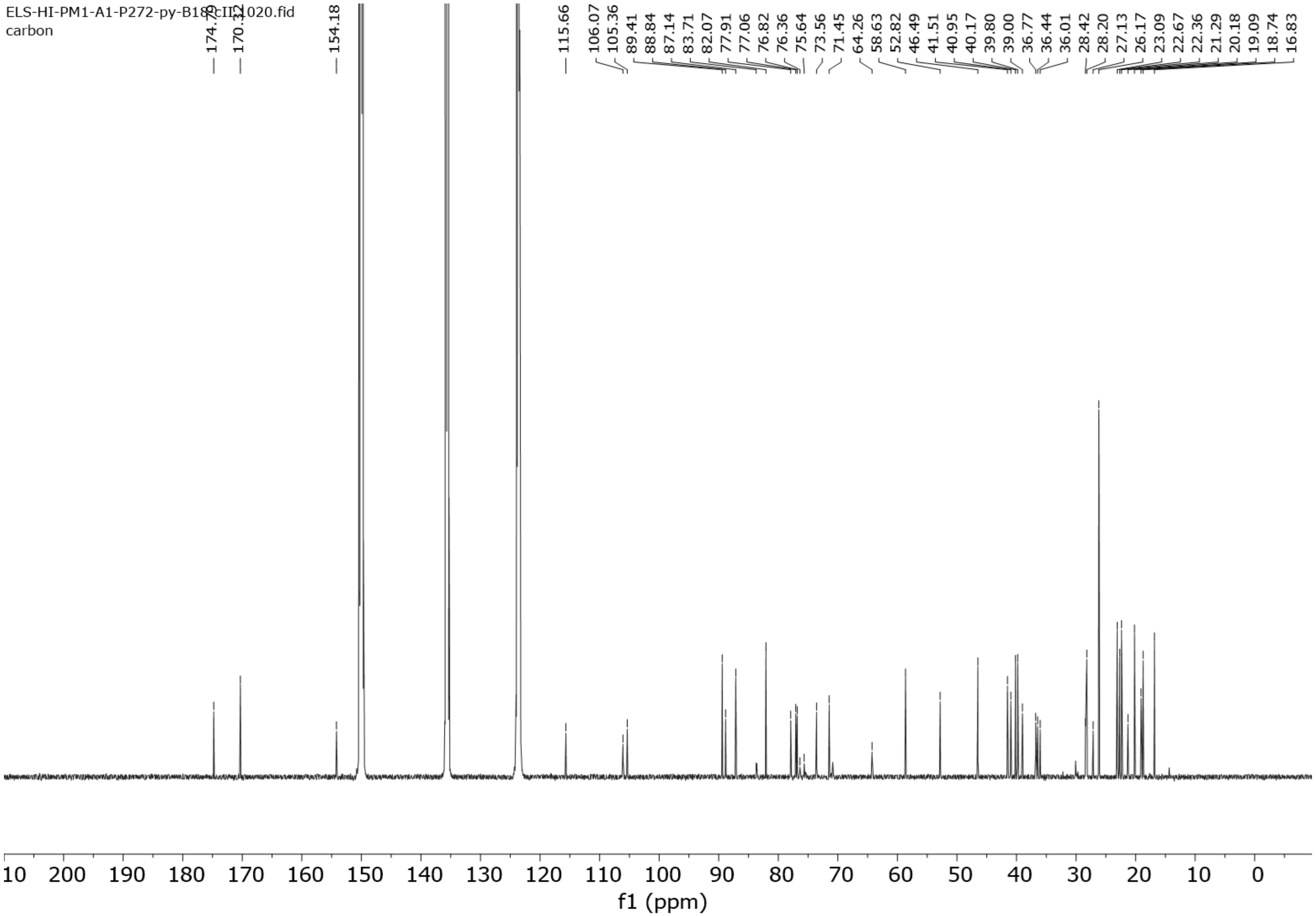

Figure S3. ^{13}**C NMR spectrum of Inornatoside A (1) (700 MHz, Pyridine-*d*$_5$)**

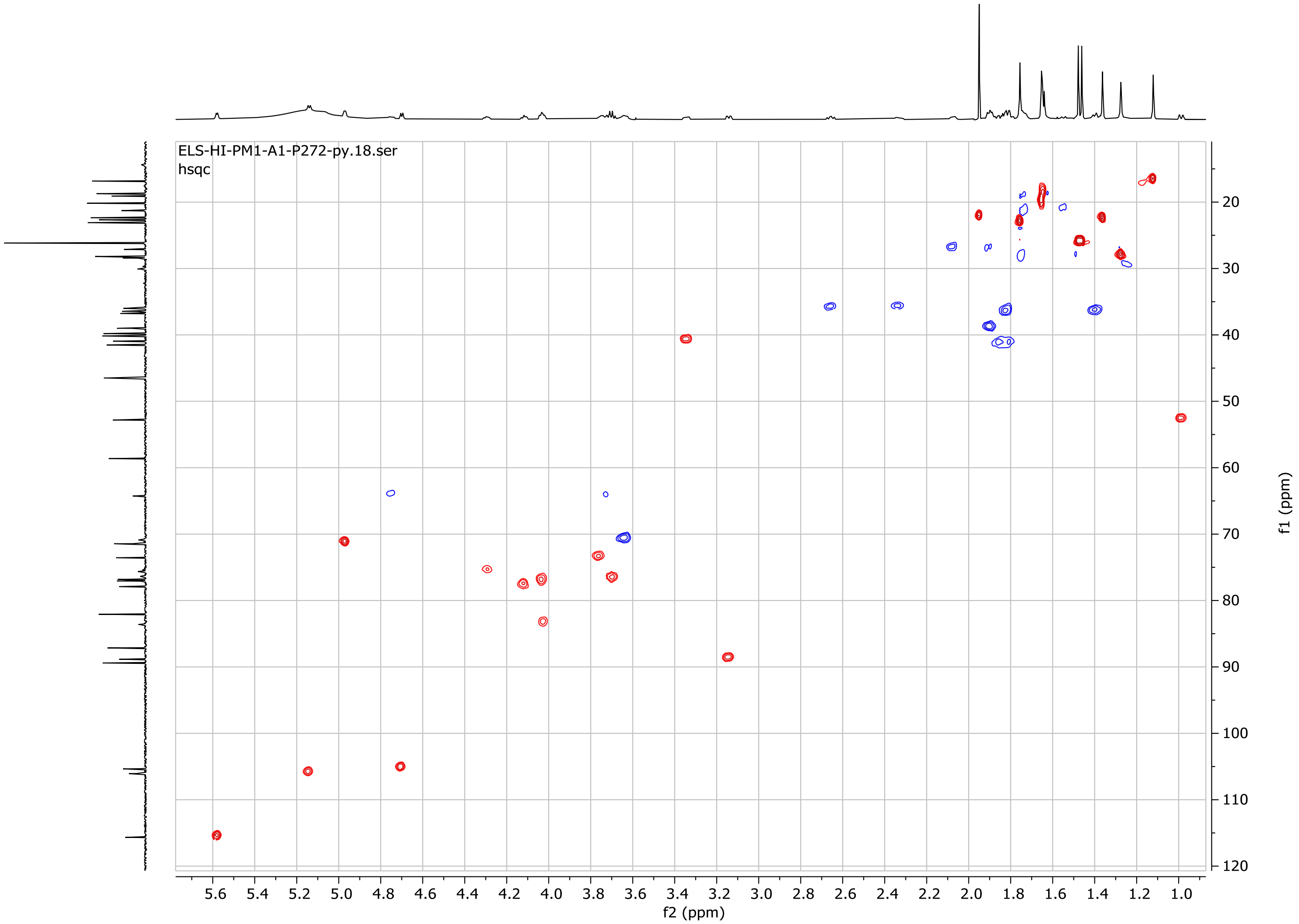

Figure S4. HSQC spectrum of Inornatoside A (1) (700 MHz, Pyridine-*d*$_5$)

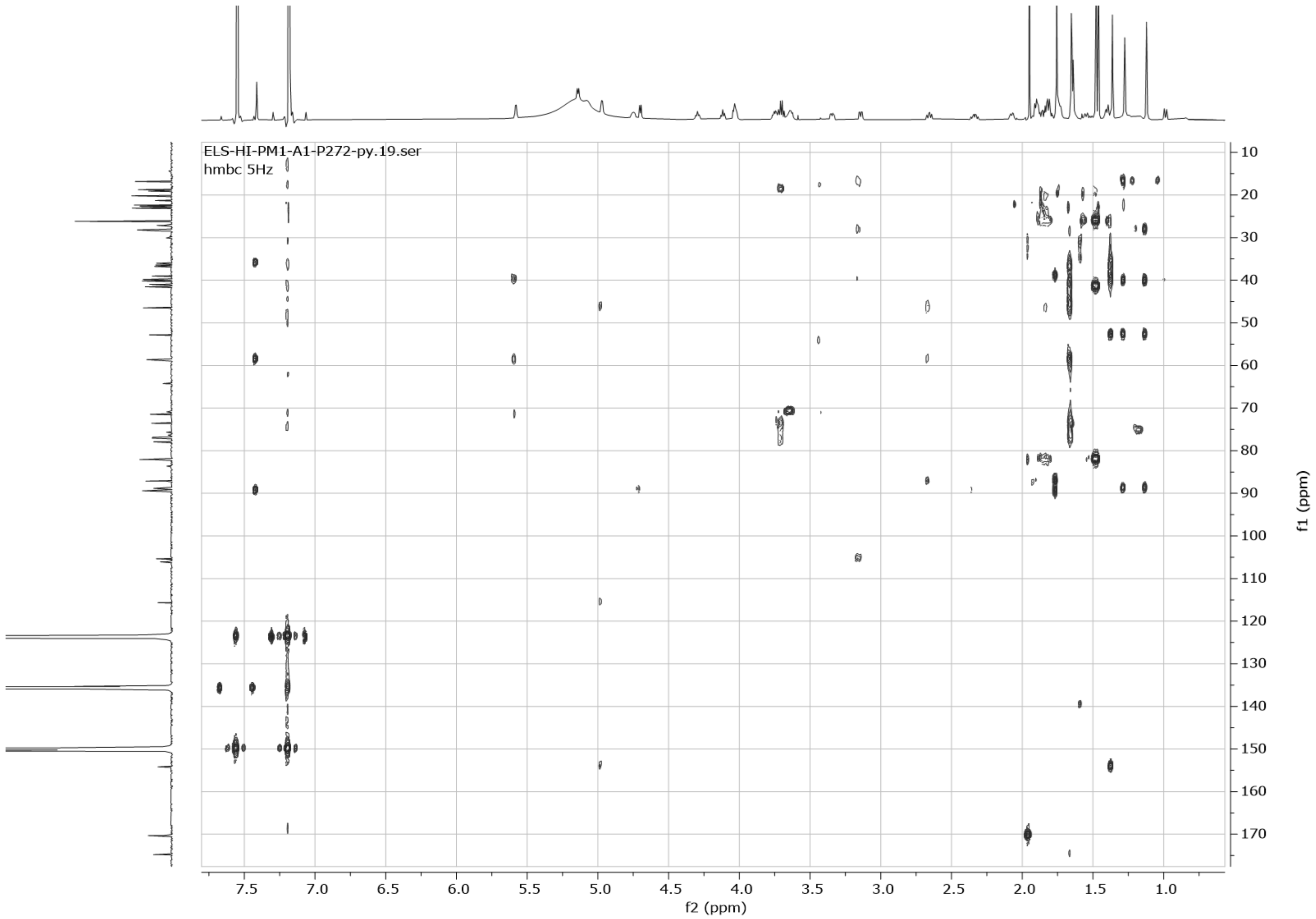

Figure S5. HMBC spectrum of Inornatoside A (1) (700 MHz, Pyridine-d_5)

Elemental Composition Report **Page 1**

Tolerance = 3.0 mDa / DBE: min = -1.5, max = 50.0
Element prediction: Off
Number of isotope peaks used for i-FIT = 3

Monoisotopic Mass, Even Electron Ions
1243 formula(e) evaluated with 4 results within limits (up to 50 closest results for each mass)
Elements Used:
C: 1-500 H: 1-1000 O: 1-100 S: 0-1
HI-PM1-A1-2-NEG 342 (6.111)
1: TOF MS ES-

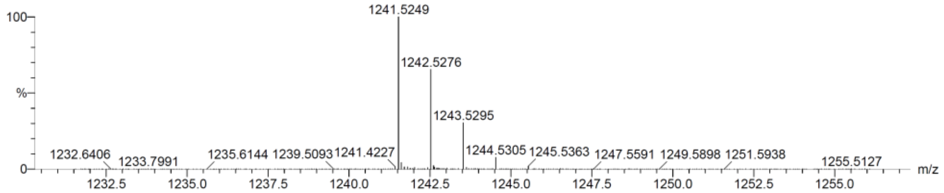

	Mass	RA	Calc. Mass	mDa	PPM	DBE	i-FIT	Norm	Conf(%)	Formula
Minimum:		80.00				-1.5				
Maximum:		100.00		3.0	10.0	50.0				
	1241.5249	100.00	1241.5261	-1.2	-1.0	12.5	478.8	0.007	99.35	C56 H89 O28 S

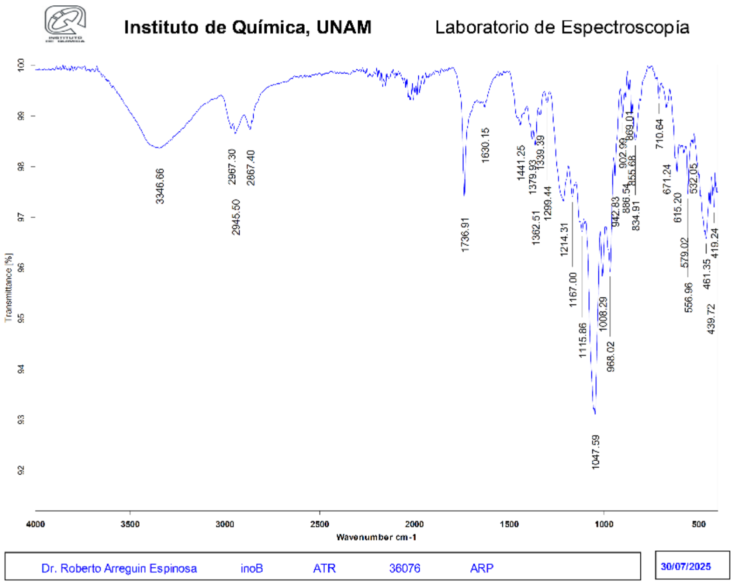

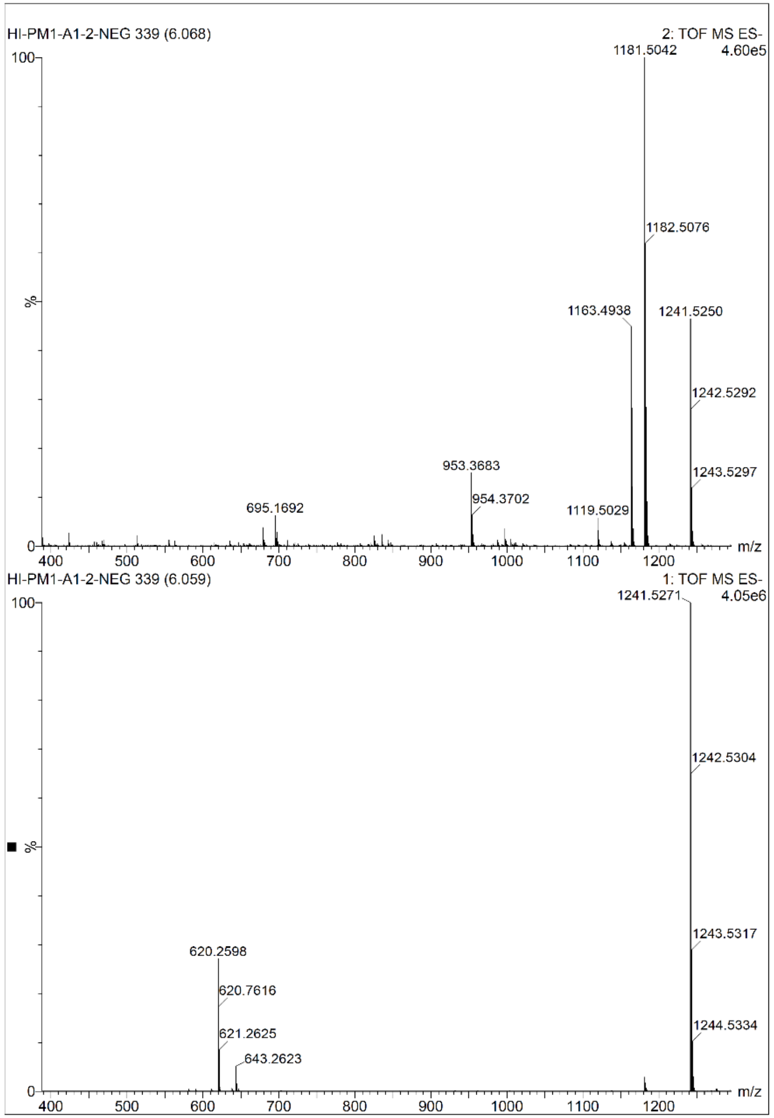

Figure S6. IR and HRESI MS^{E} (negative mode) of Inornatoside B (2).

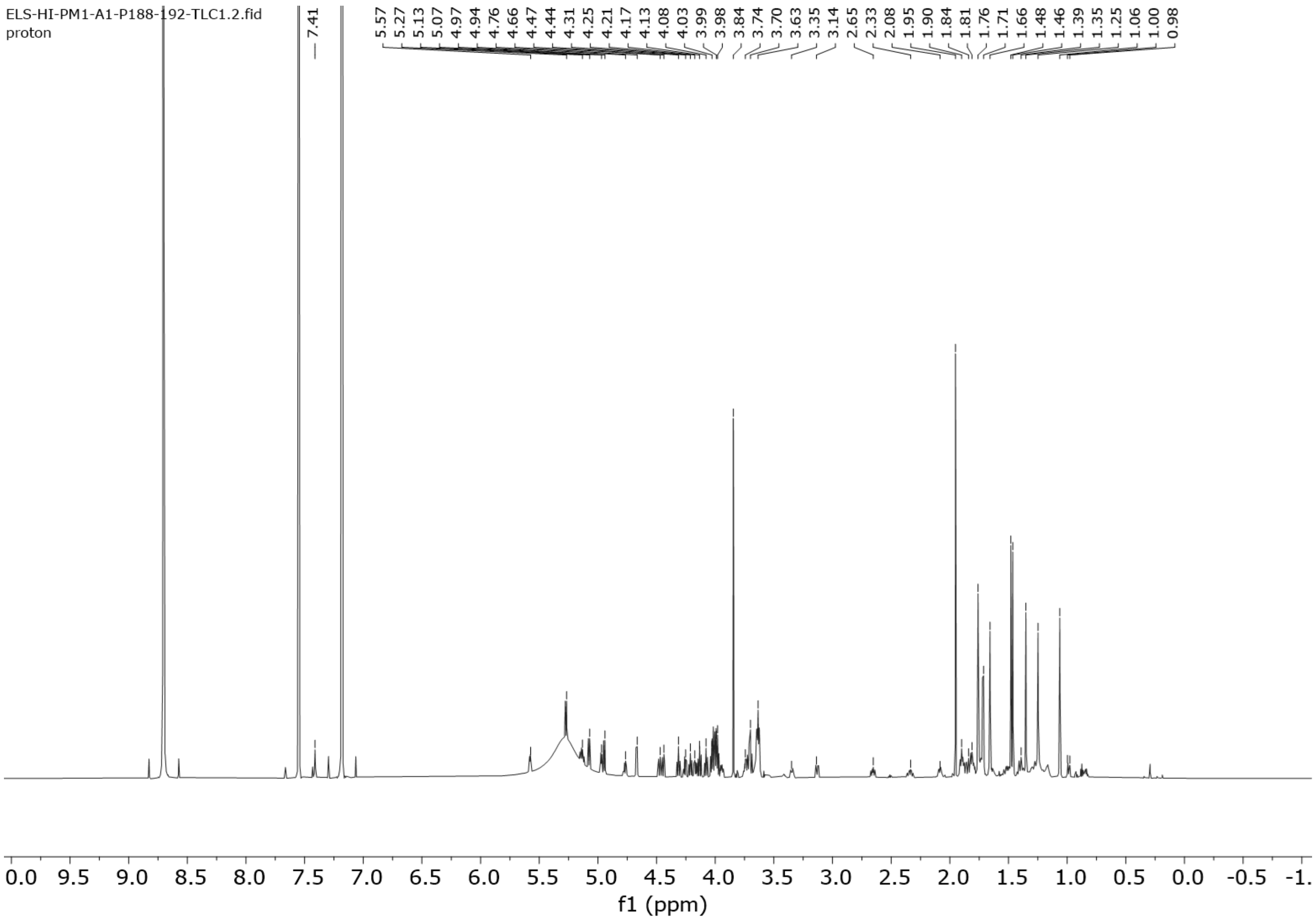

Figure S7. **^{1}H NMR spectrum of Inornatoside B (2) (700 MHz, Pyridine-d_5)**

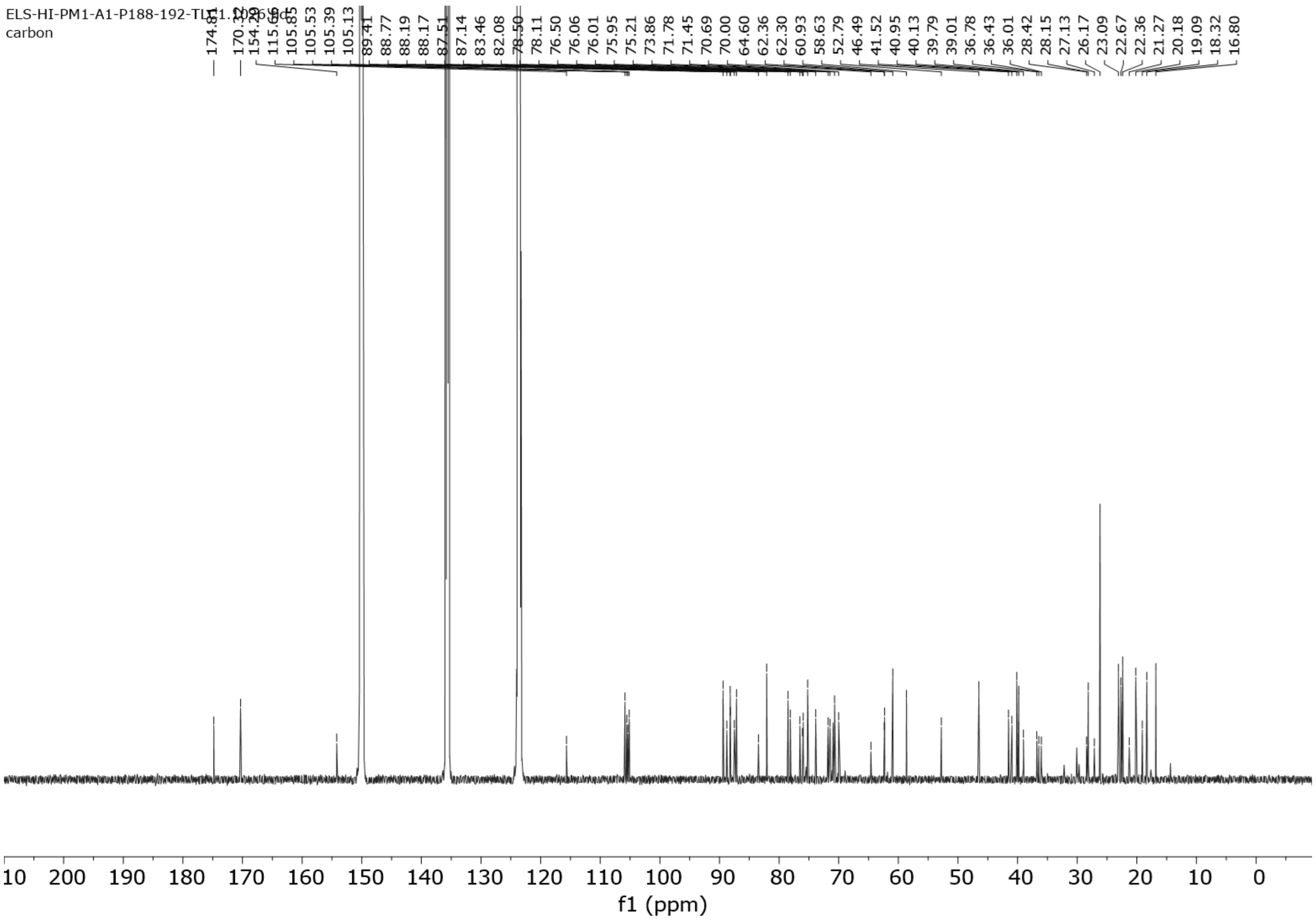

Figure S8. **^{13}C NMR spectrum of Inornatoside B (2) (700 MHz, Pyridine-d_5)**

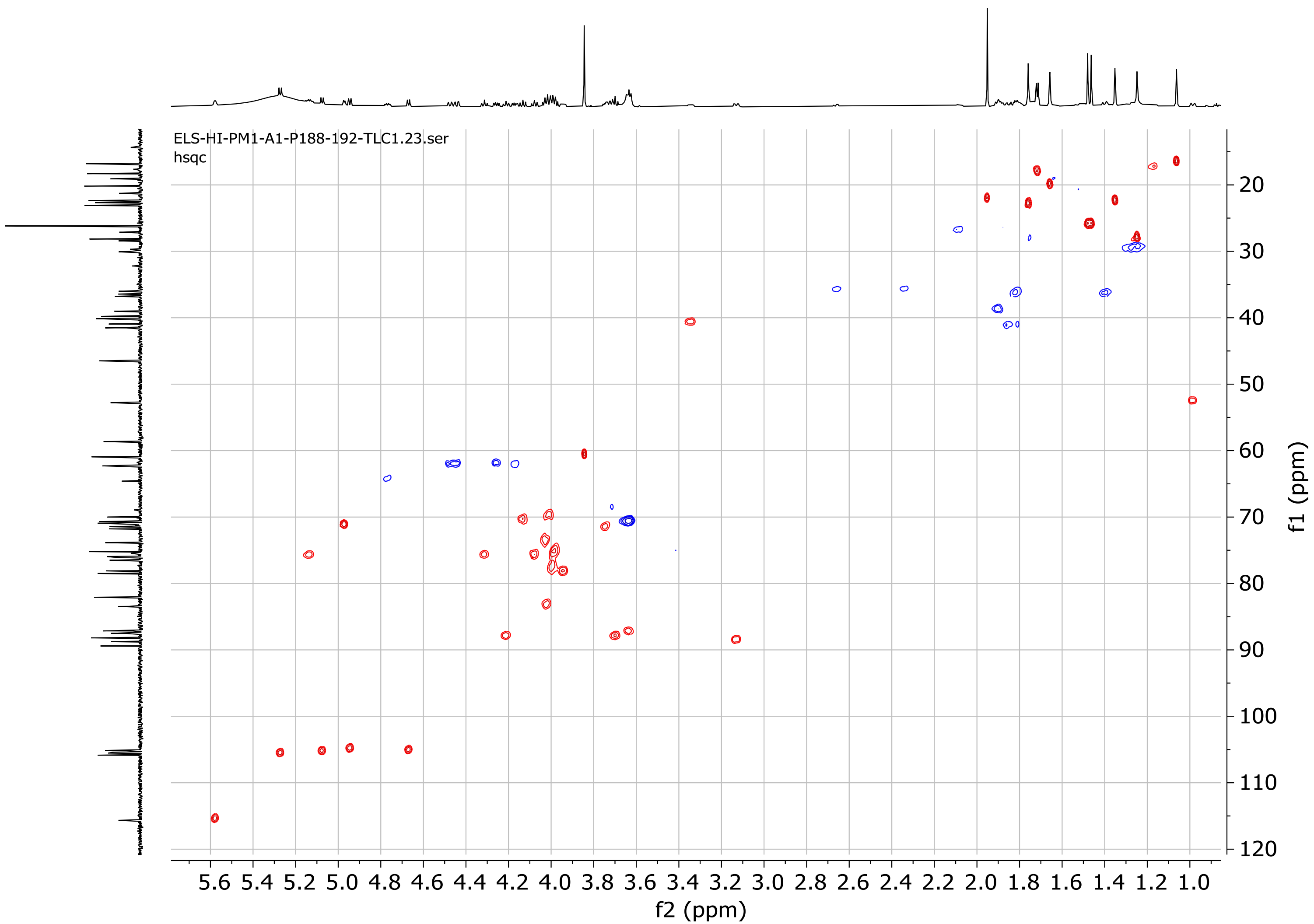

Figure S9. HSQC spectrum of Inornatoside B (2) (700 MHz, Pyridine-*d*$_5$)

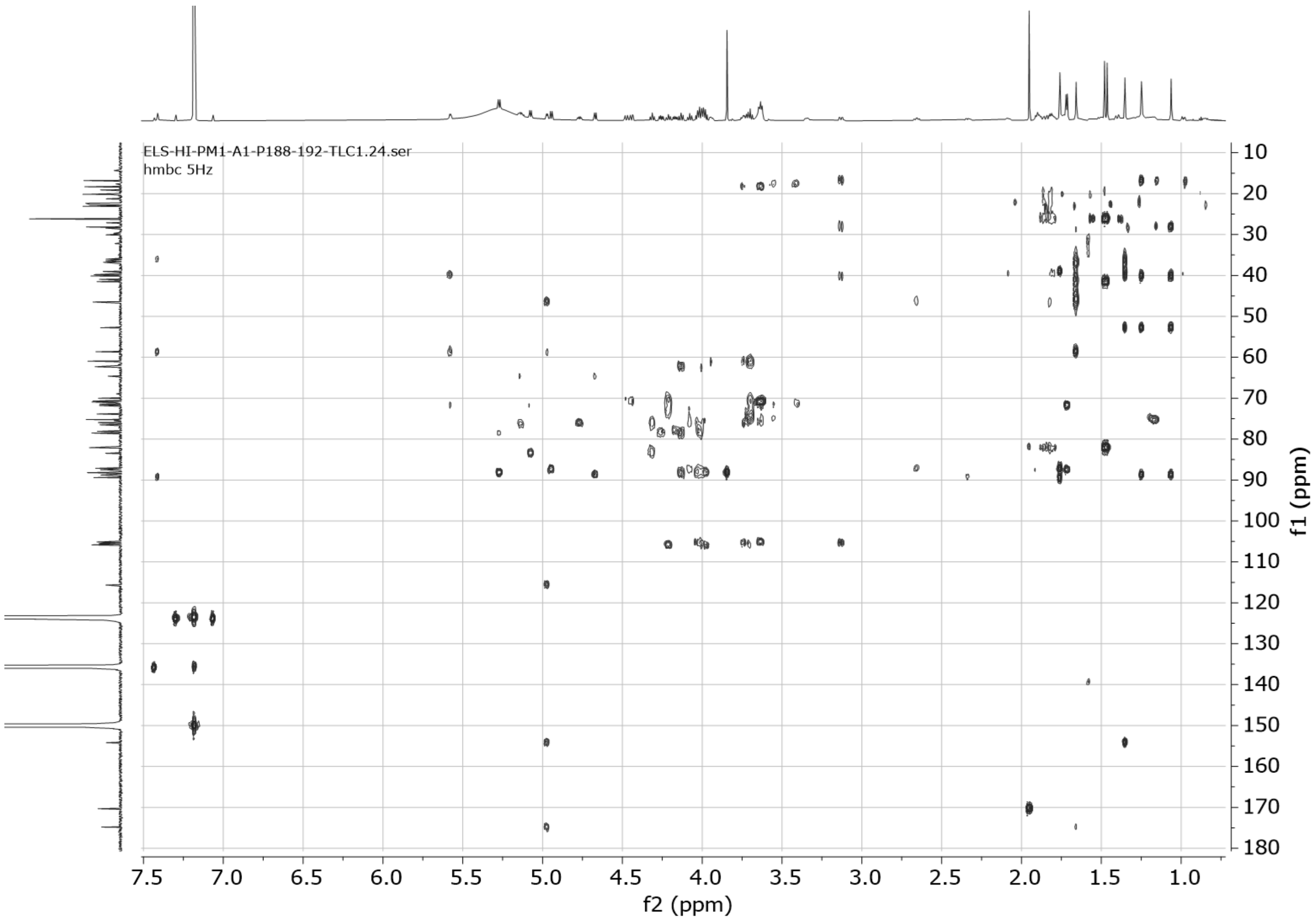

Figure S10. HMBC spectrum of Inornatoside B (2) (700 MHz, Pyridine-d_5)

Elemental Composition Report **Page 1**

Tolerance = 3.0 mDa / DBE: min = -1.5, max = 50.0
Element prediction: Off
Number of isotope peaks used for i-FIT = 3

Monoisotopic Mass, Even Electron Ions
618 formula(e) evaluated with 3 results within limits (up to 50 closest results for each mass)
Elements Used:
C: 1-500 H: 1-1000 O: 1-100 S: 0-1

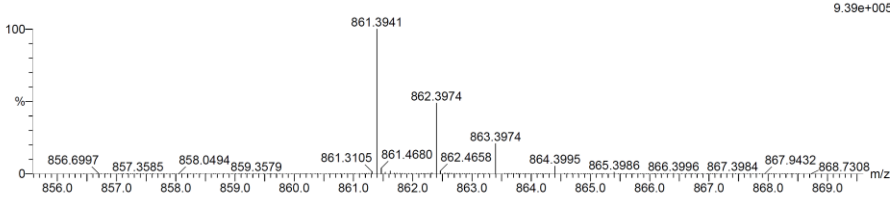

Minimum:	80.00				-1.5				
Maximum:	100.00		3.0	10.0	50.0				
Mass	RA	Calc. Mass	mDa	PPM	DBE	i-FIT	Norm	Conf(%)	Formula
861.3941	100.00	861.3942	-0.1	-0.1	9.5	691.3	0.000	99.99	C41 H65 O17 S

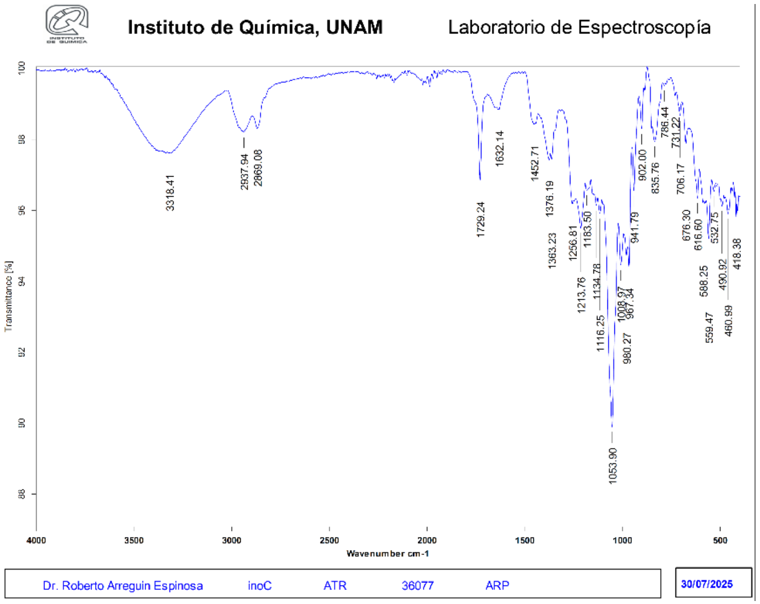

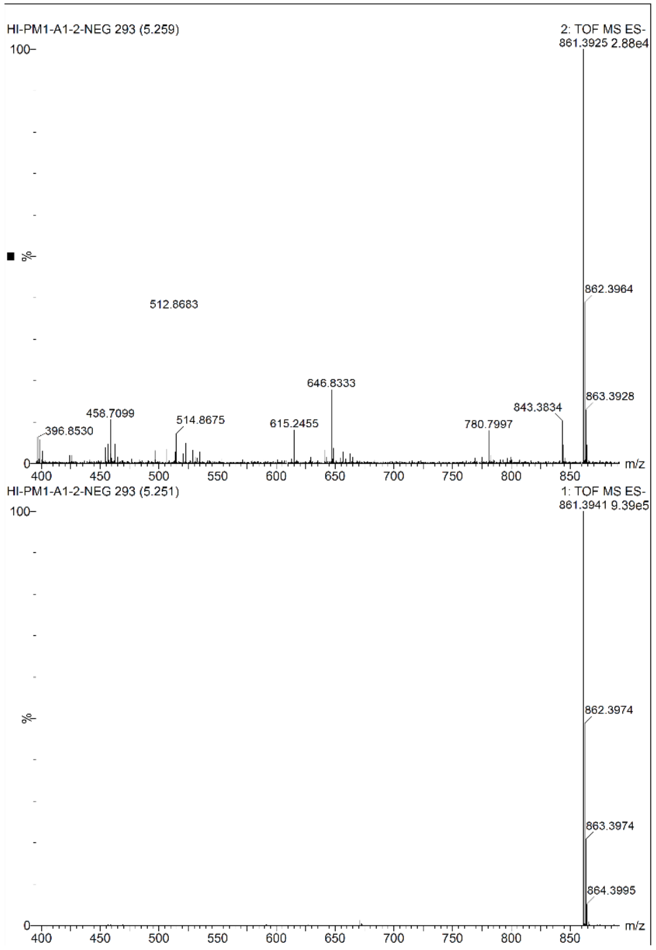

Figure S11. IR and HRESI MS^{E} (negative mode) of Inornatoside C (3).

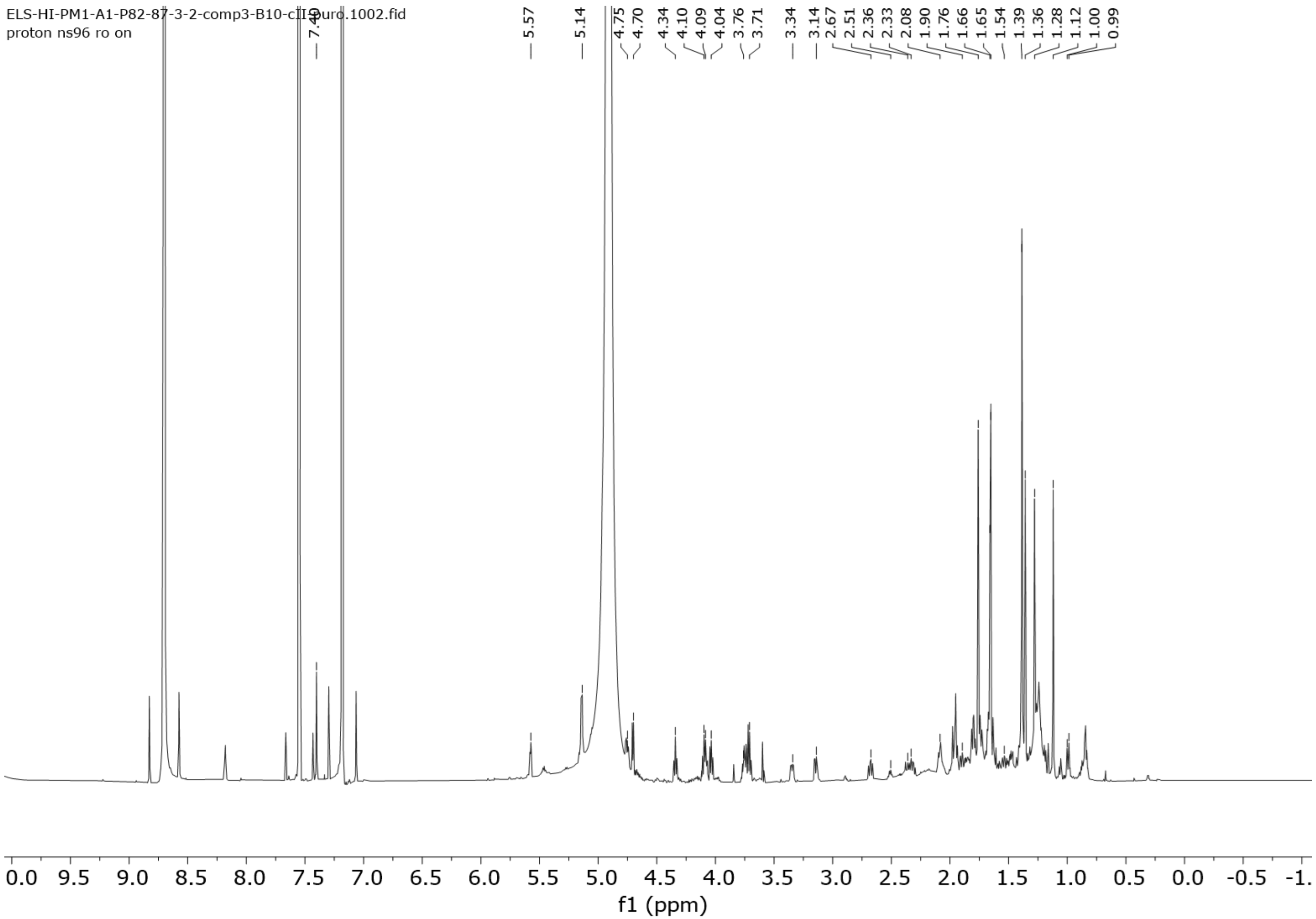

Figure S12. ^{1}H NMR spectrum of Inornatoside C (3) (700 MHz, Pyridine-d_5)

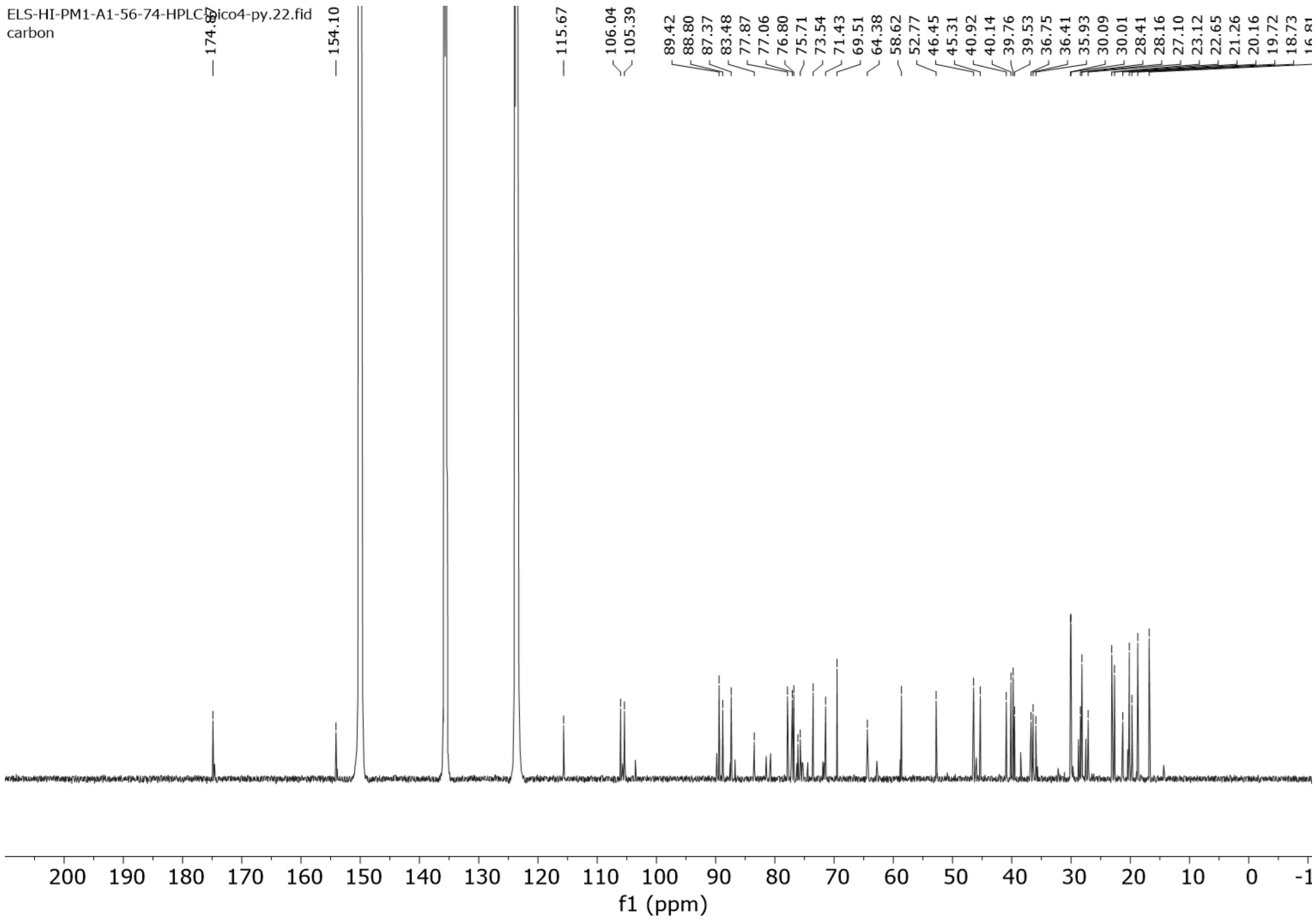

Figure S13. ^{13}**C NMR spectrum of Inornatoside C (3) (700 MHz, Pyridine-*d*$_5$)**

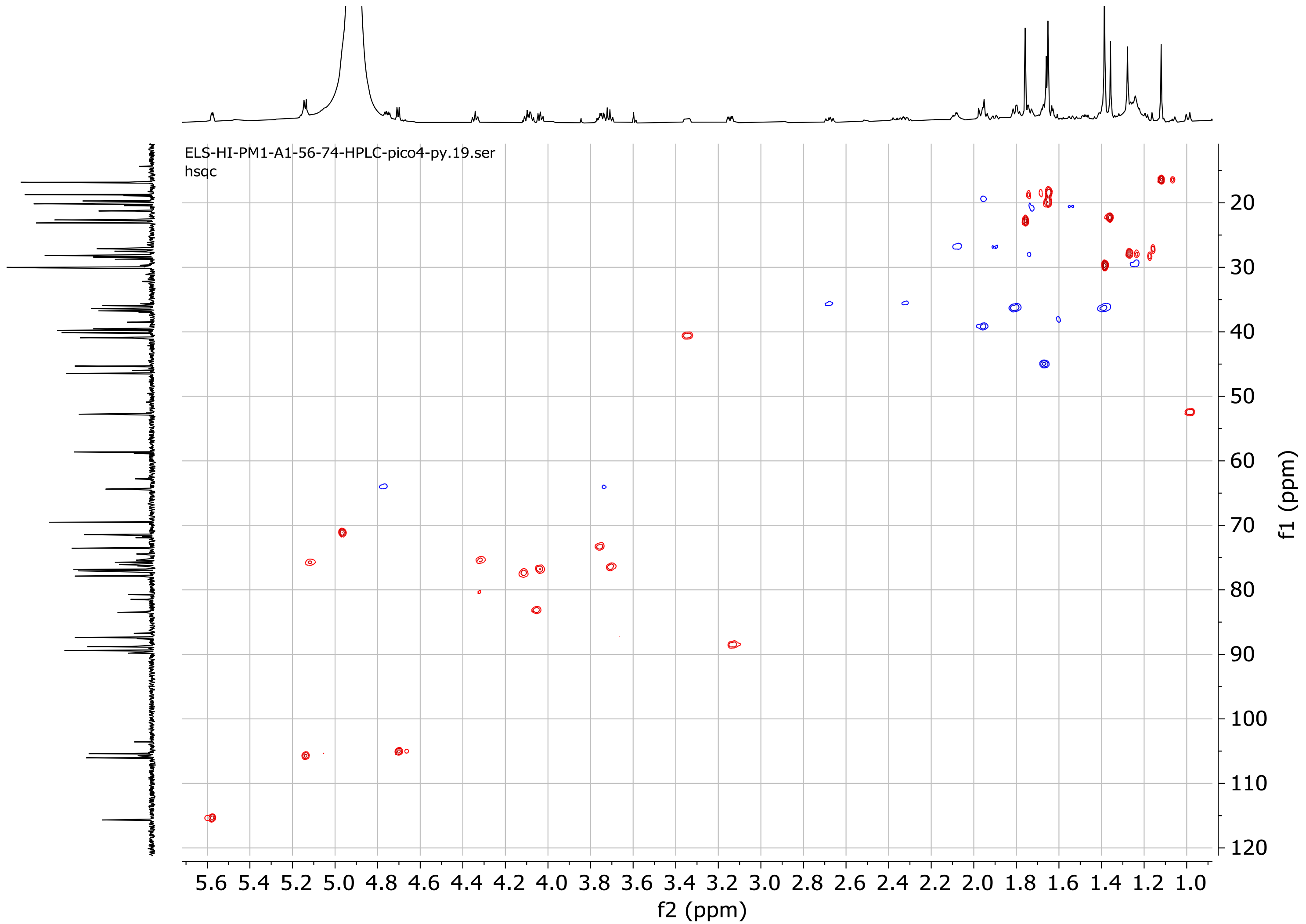

Figure S14. HSQC spectrum of Inornatoside C (3) (700 MHz, Pyridine-d_5)

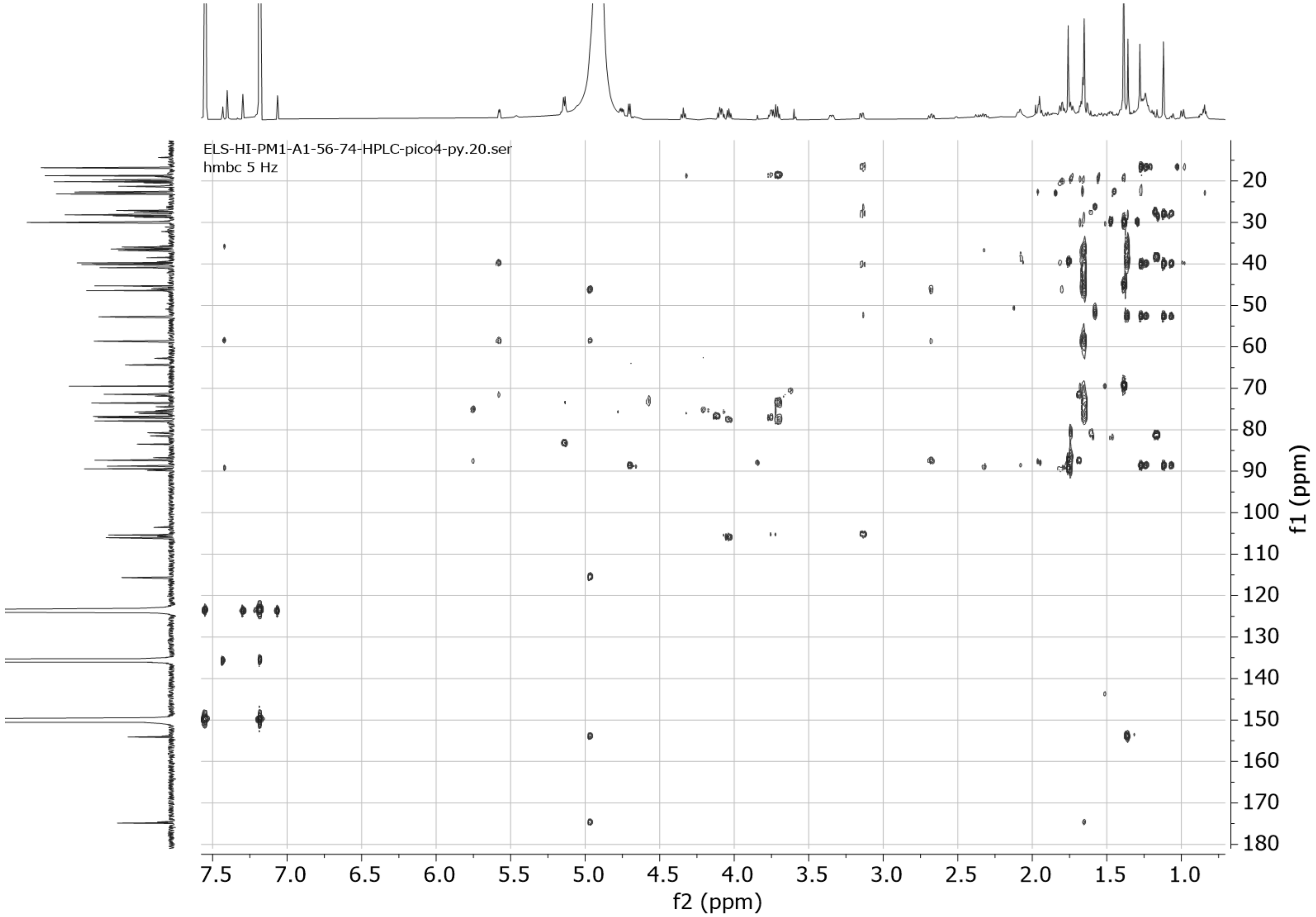

Figure S15. HMBC spectrum of Inornatoside C (3) (700 MHz, Pyridine-d_5)

Elemental Composition Report

Tolerance = 3.0 mDa / DBE: min = -1.5, max = 50.0
Element prediction: Off
Number of isotope peaks used for i-FIT = 3

Monoisotopic Mass, Even Electron Ions
634 formula(e) evaluated with 3 results within limits (up to 50 closest results for each mass)
Elements Used:
C: 1-500 H: 1-1000 O: 1-100 S: 0-1
HI-PM1-A1-82-87-Pico5-NEG 389 (6.965)
2: TOF MS ES-

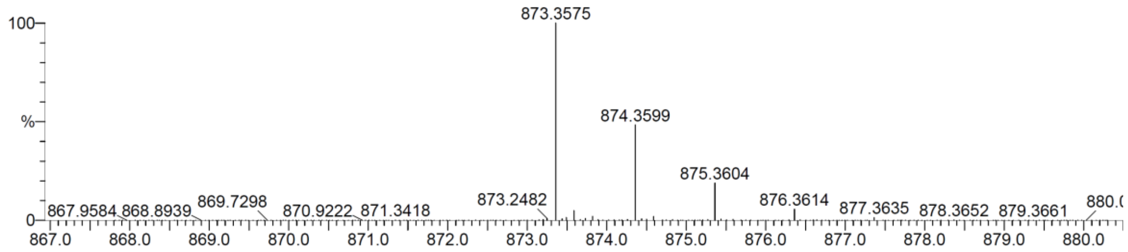

Minimum:	80.00				-1.5				
Maximum:	100.00		3.0	10.0	50.0				
Mass	RA	Calc. Mass	mDa	PPM	DBE	i-FIT	Norm	Conf(%)	Formula
873.3575	100.00	873.3579	-0.4	-0.5	11.5	693.5	0.000	100.00	C41 H61 O18 S

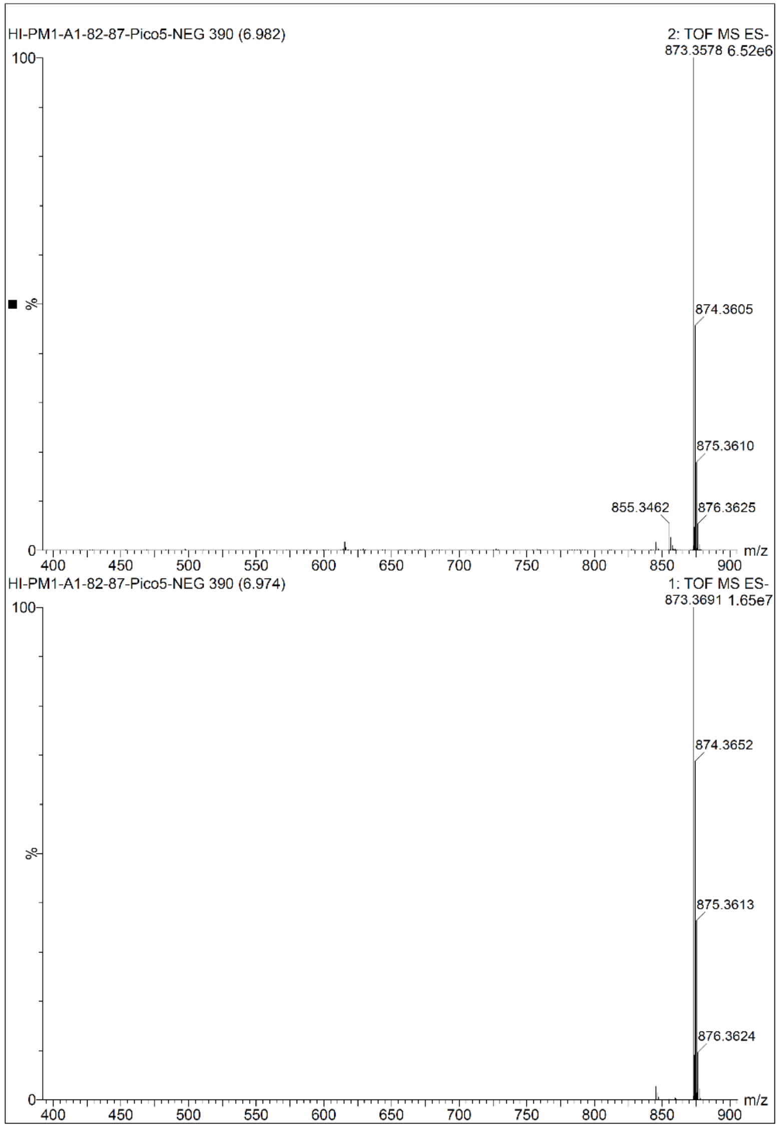

Figure S16. HRESI MS^{E} (negative mode) of Inornatoside D (4).

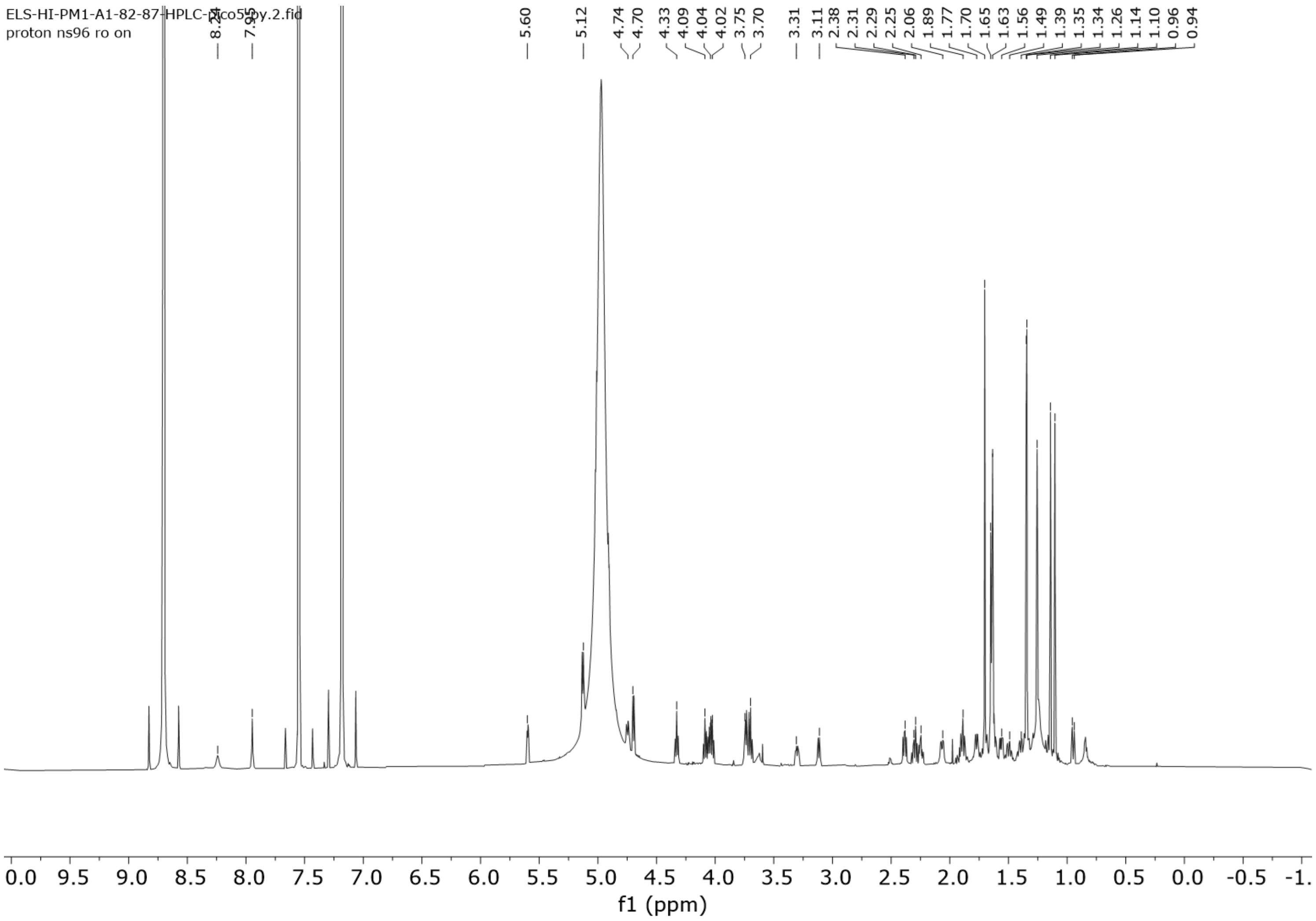

Figure S17. **^{1}H NMR spectrum of Inornatoside D (4) (700 MHz, Pyridine-d_5)**

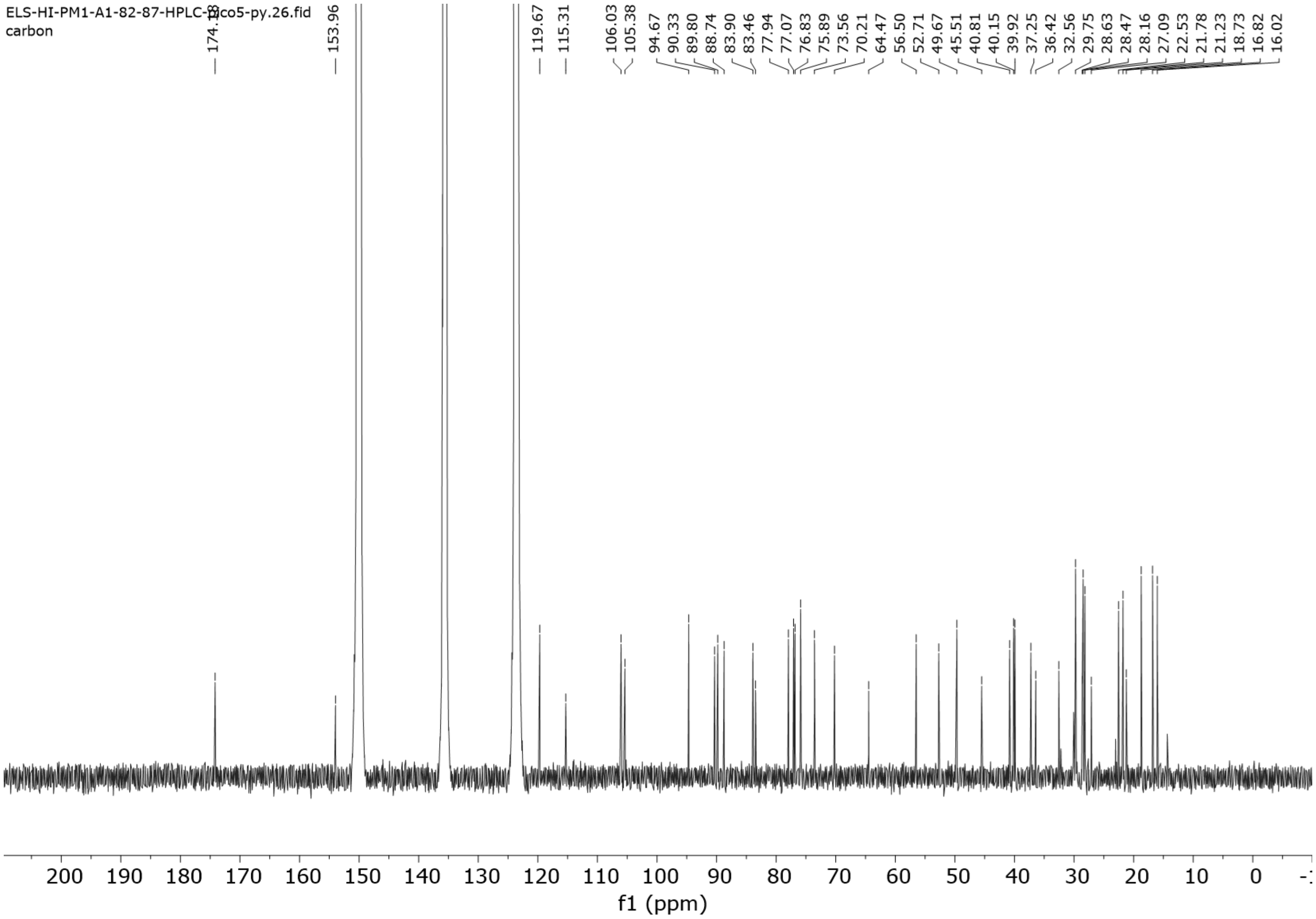

Figure S18. ^{13}C **NMR spectrum of Inornatoside D (4) (700 MHz, Pyridine-*d*$_5$)**

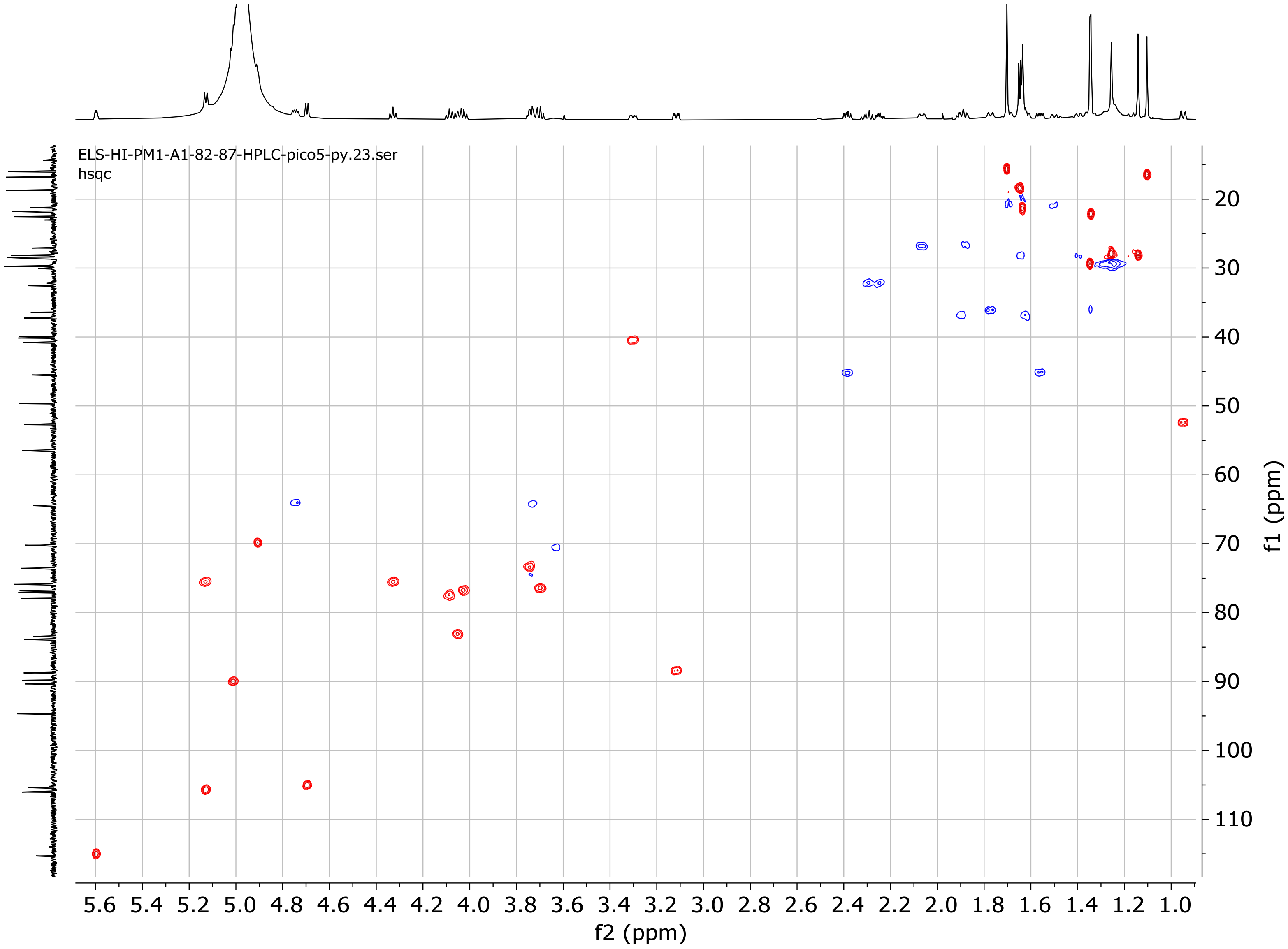

Figure S19. HSQC spectrum of Inornatoside D (4) (700 MHz, Pyridine-*d*$_5$)

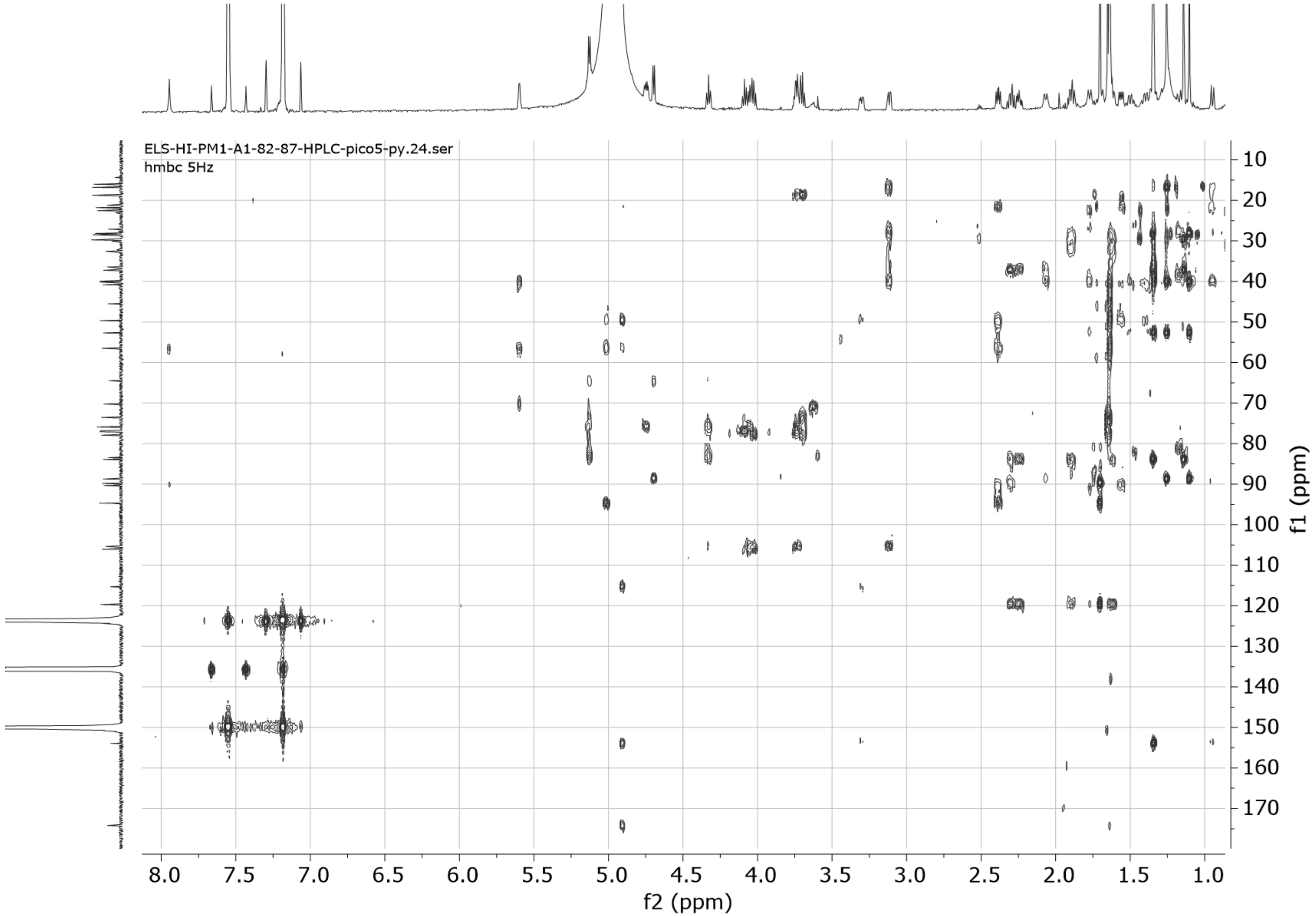

Figure S20. HMBC spectrum of Inornatoside D (4) (700 MHz, Pyridine-*d*$_5$)

Elemental Composition Report **Page 1**

Tolerance = 3.0 mDa / DBE: min = -1.5, max = 50.0
Element prediction: Off
Number of isotope peaks used for i-FIT = 3

Monoisotopic Mass, Even Electron Ions
528 formula(e) evaluated with 3 results within limits (up to 50 closest results for each mass)
Elements Used:
C: 1-500 H: 1-1000 O: 1-100 S: 0-1
HI-PM1-A1-44-55-1a-3-NEG 251 (4.500)
2: TOF MS ES-

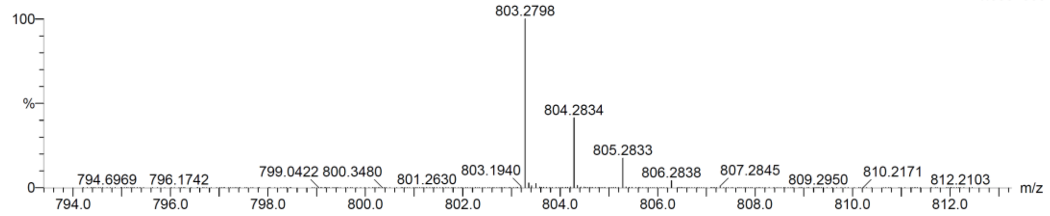

Minimum:	80.00				-1.5				
Maximum:	100.00		3.0	10.0	50.0				
Mass	RA	Calc. Mass	mDa	PPM	DBE	i-FIT	Norm	Conf(%)	Formula
803.2798	100.00	803.2796	0.2	0.2	11.5	656.5	0.000	100.00	C36 H51 O18 S

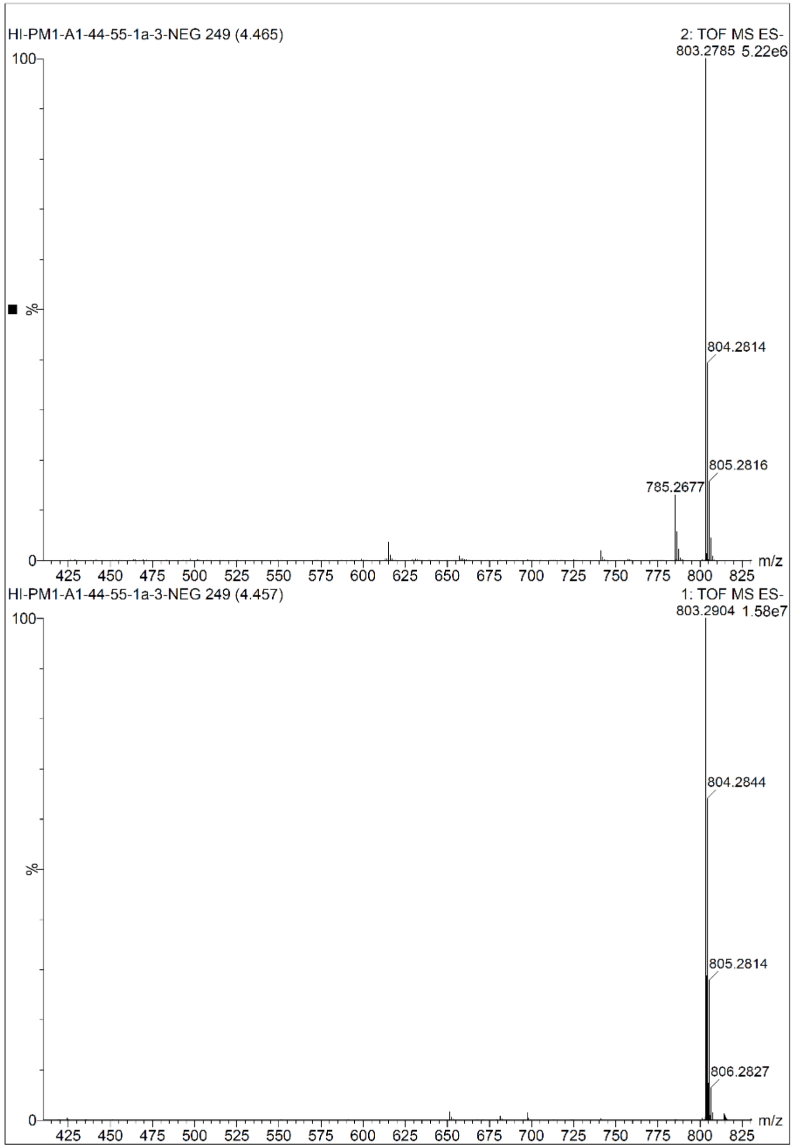

Figure S21. HRESI MS^E (negative mode) of Inornatoside E (5).

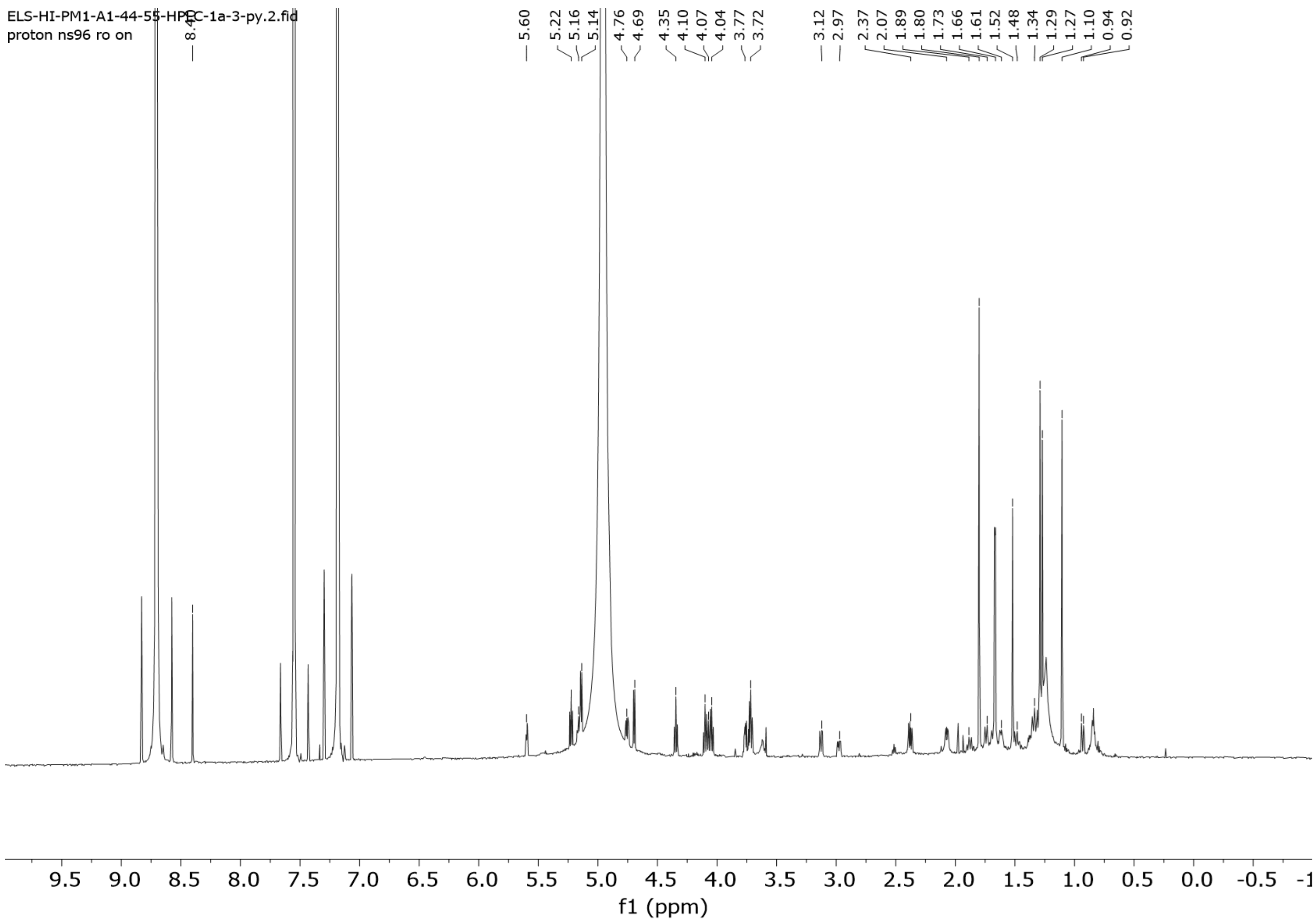

Figure S22. **^{1}H NMR spectrum of Inornatoside E (5) (700 MHz, Pyridine-*d_5*)**

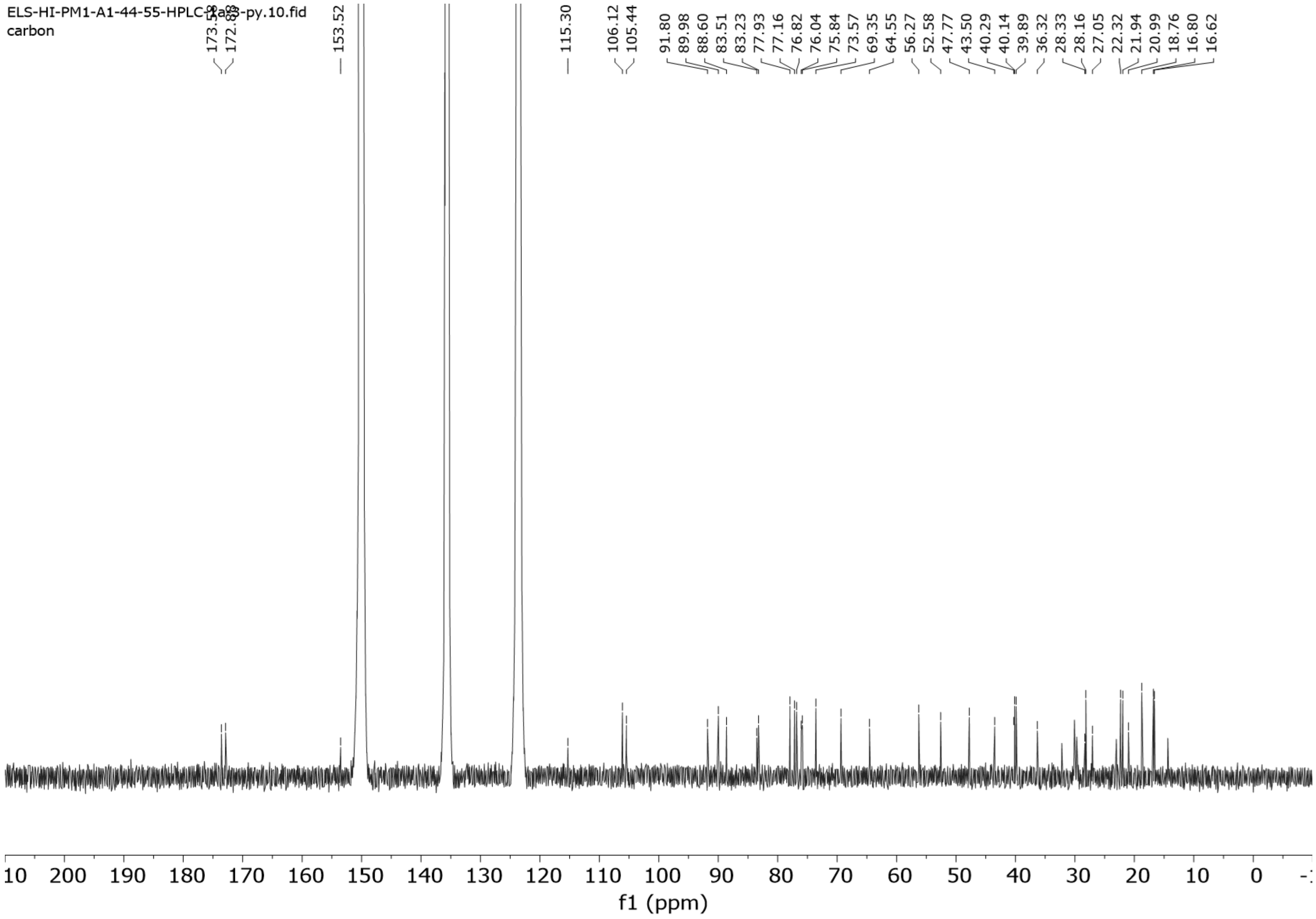

Figure S23. ^{13}**C NMR spectrum of Inornatoside E (5) (700 MHz, Pyridine-*d*$_5$)**

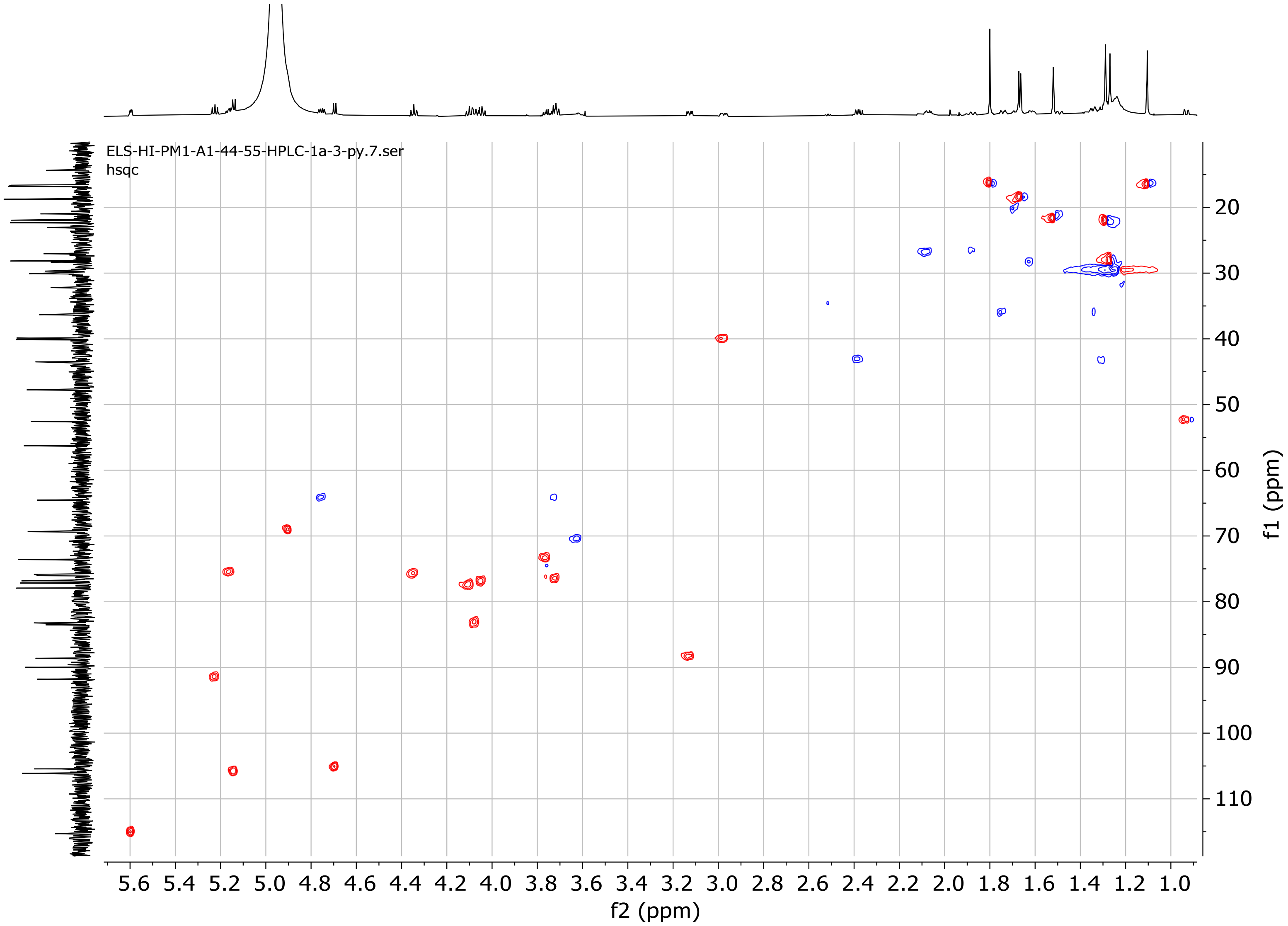

Figure S24. HSQC spectrum of Inornatoside E (5) (700 MHz, Pyridine-d_5)

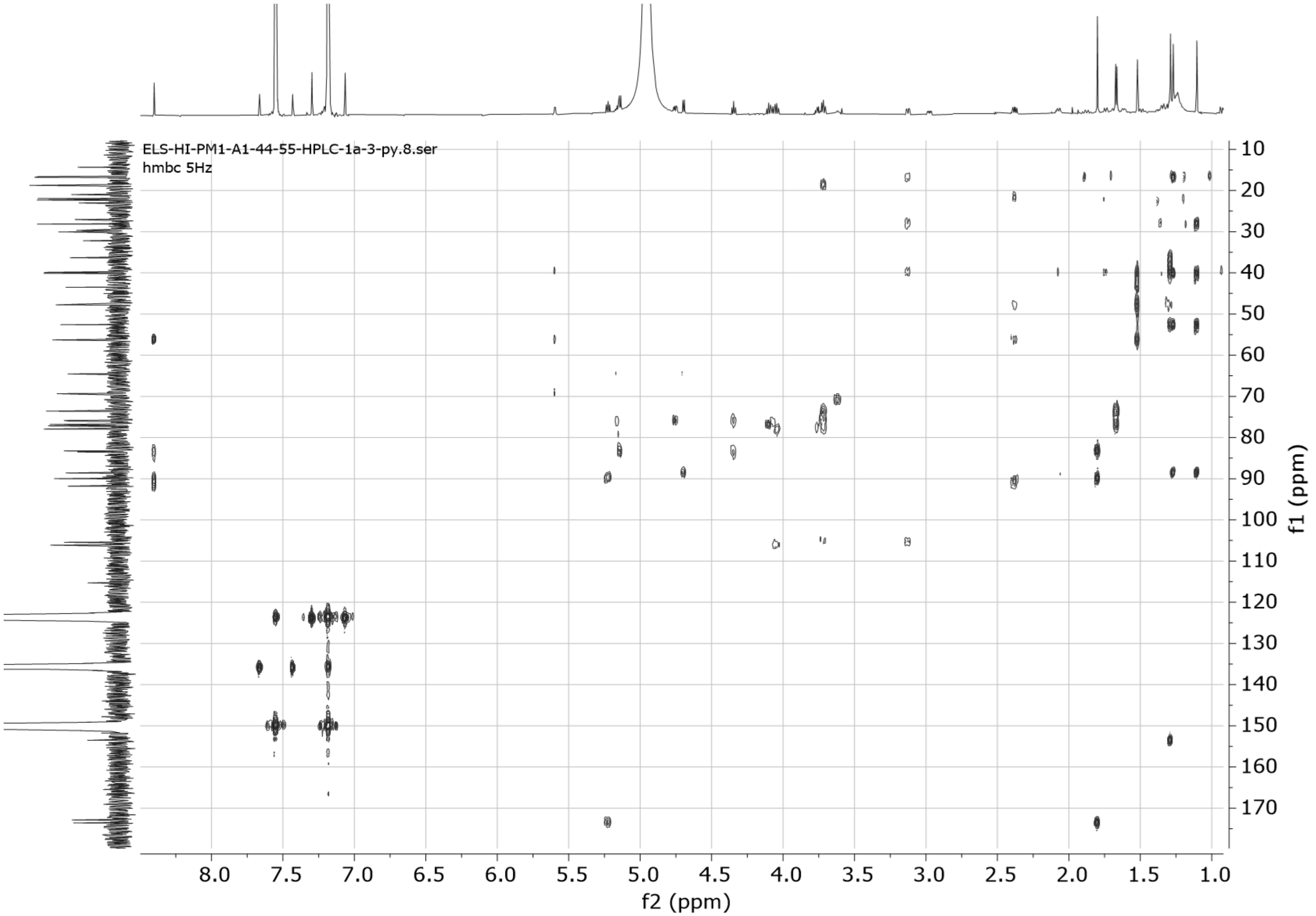

Figure S25. HMBC spectrum of Inornatoside E (5) (700 MHz, Pyridine-d_5)

Elemental Composition Report **Page 1**

Tolerance = 3.0 mDa / DBE: min = -1.5, max = 50.0
Element prediction: Off
Number of isotope peaks used for i-FIT = 3

Monoisotopic Mass, Even Electron Ions
597 formula(e) evaluated with 3 results within limits (up to 50 closest results for each mass)
Elements Used:
C: 1-500 H: 1-1000 O: 1-100 S: 0-1

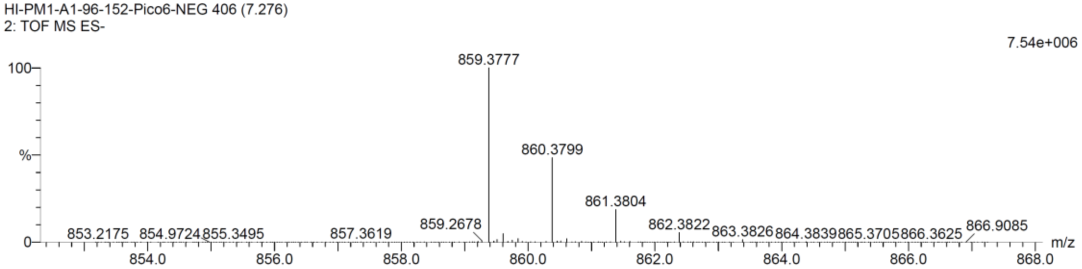

Minimum:	80.00				-1.5				
Maximum:	100.00		3.0	10.0	50.0				
Mass	RA	Calc. Mass	mDa	PPM	DBE	i-FIT	Norm	Conf(%)	Formula
859.3777	100.00	859.3786	-0.9	-1.0	10.5	667.2	0.001	99.91	C41 H63 O17 S

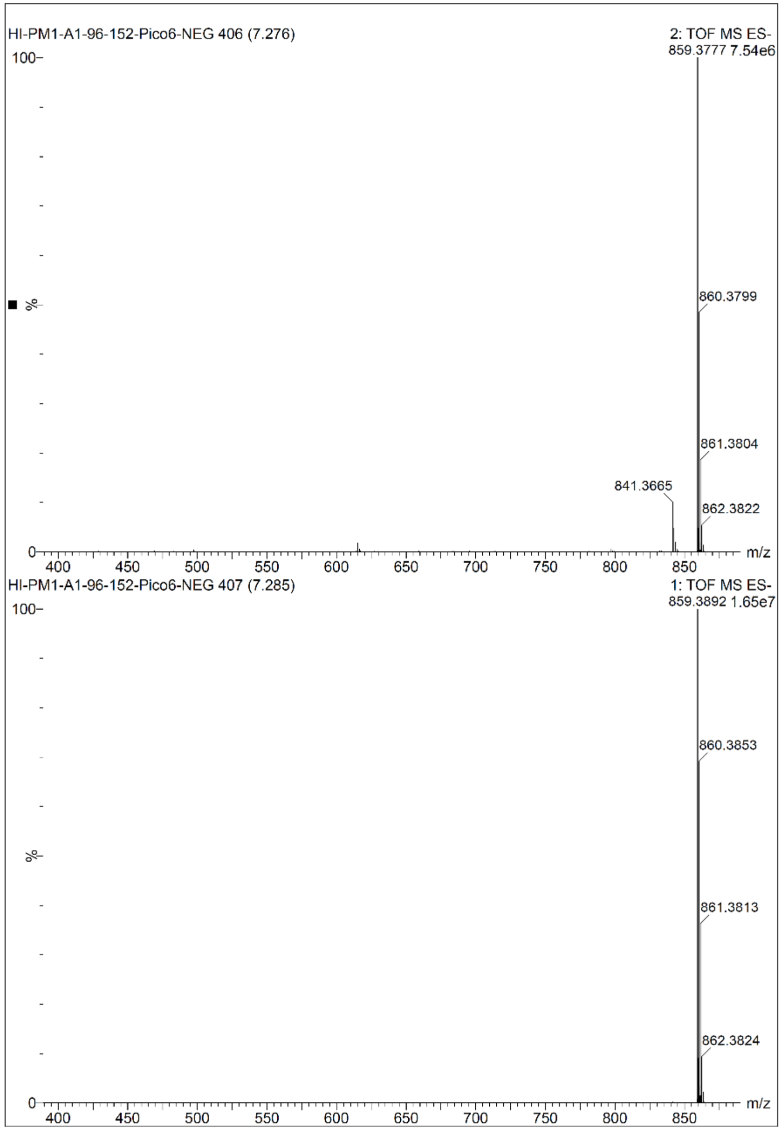

Figure S26. HRESI MS^E (negative mode) of (22*R*)-Holothurin B (6).

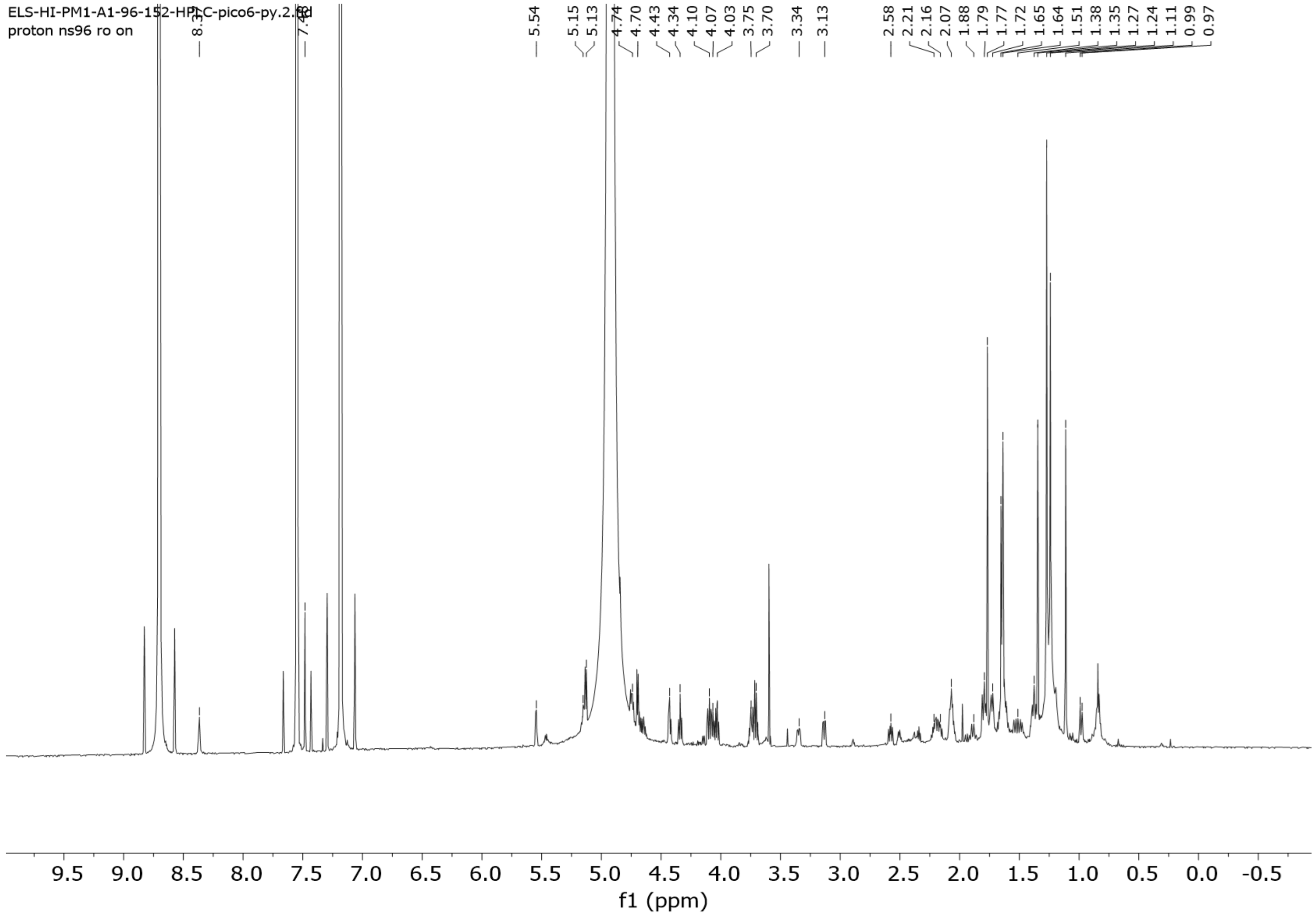

Figure S27. ¹H NMR spectrum of (22*R*)-Holothurin B (6). (700 MHz, Pyridine-*d*$_5$)

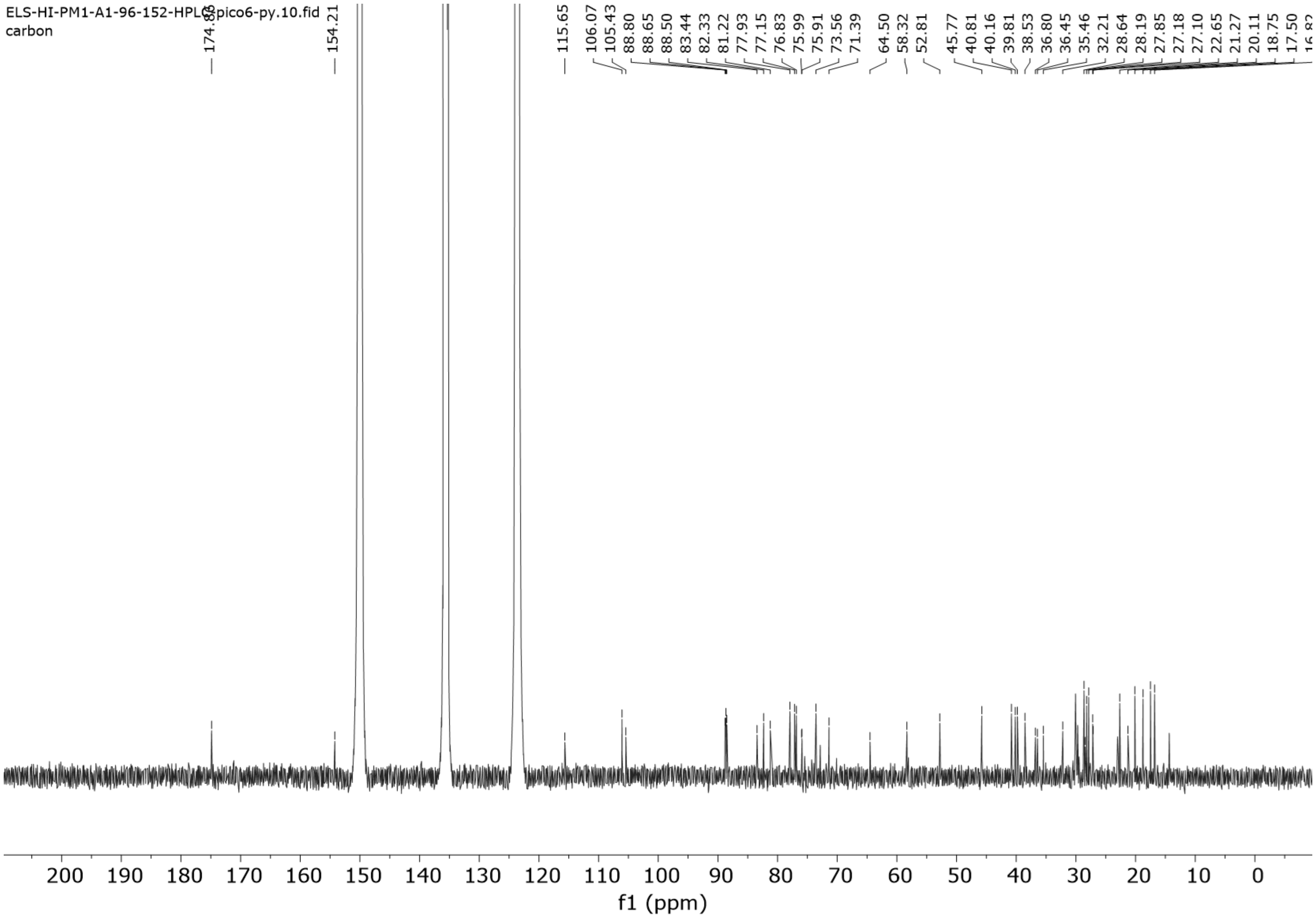

Figure S28. ^{13}**C NMR spectrum of (22*R*)-Holothurin B (6). (700 MHz, Pyridine-*d*$_5$)**

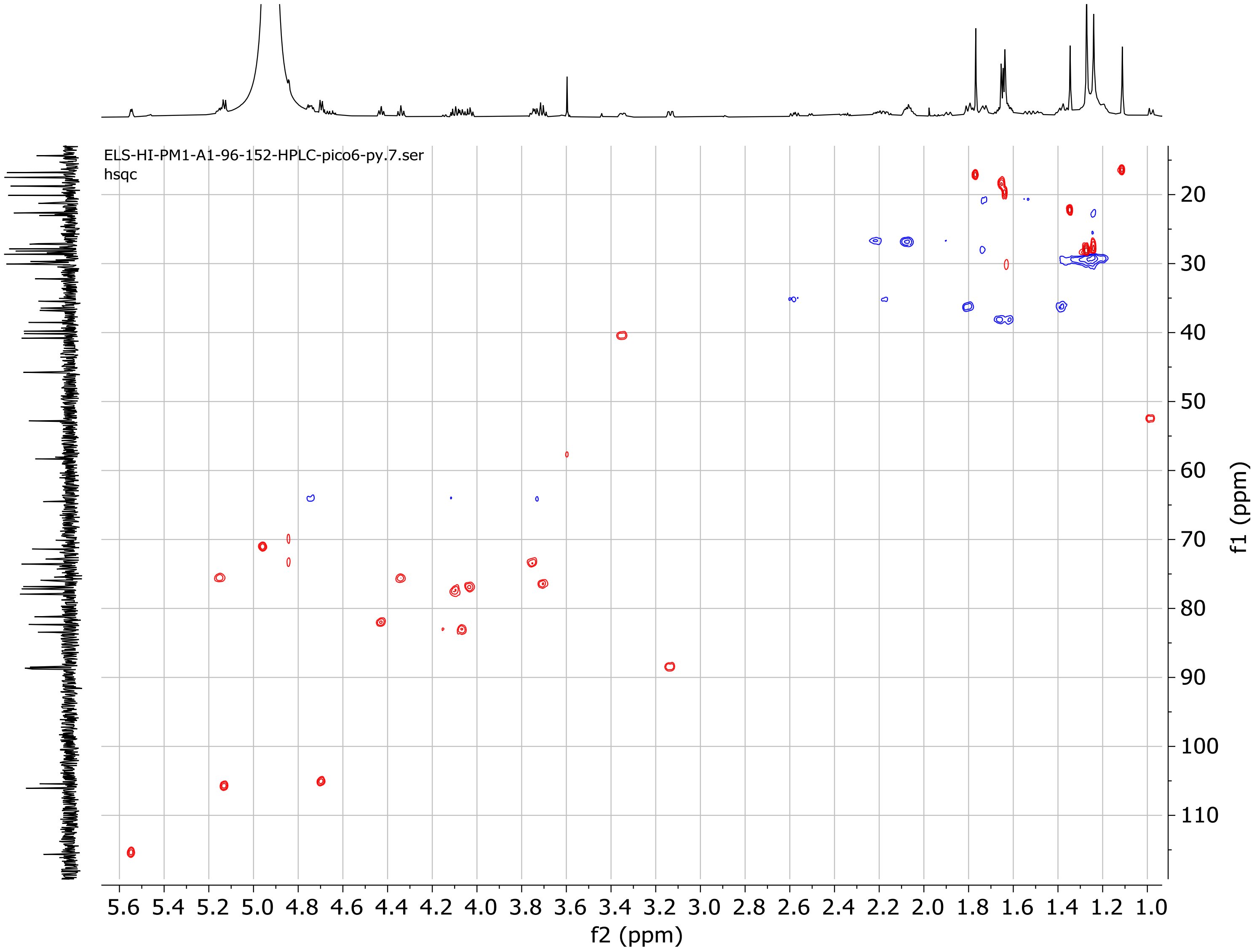

Figure S29. HSQC spectrum of (22*R*)-Holothurin B (6). (700 MHz, Pyridine-*d*$_5$)

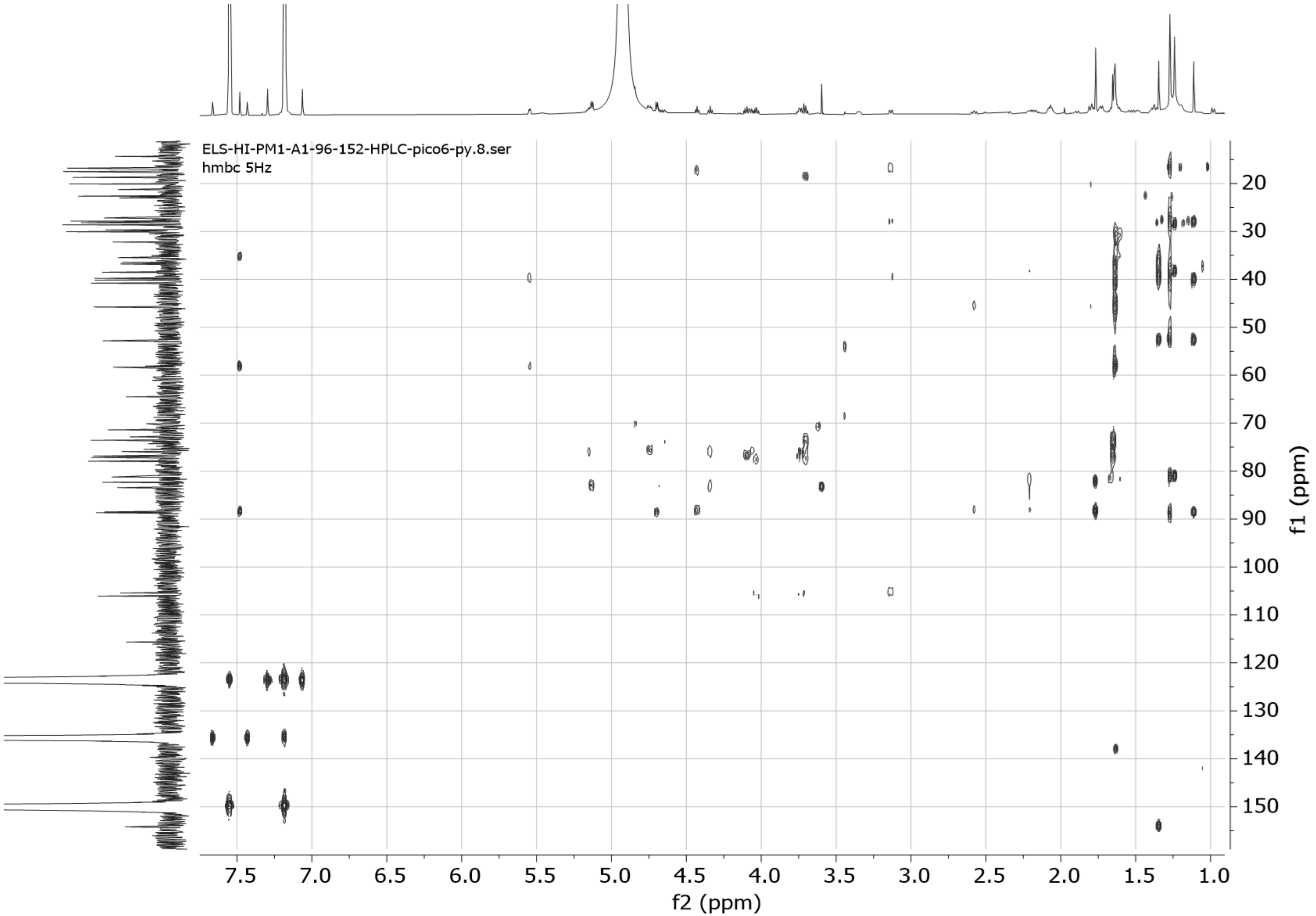

Figure S30. HMBC spectrum of (22*R*)-Holothurin B (6). (700 MHz, Pyridine-d_5)

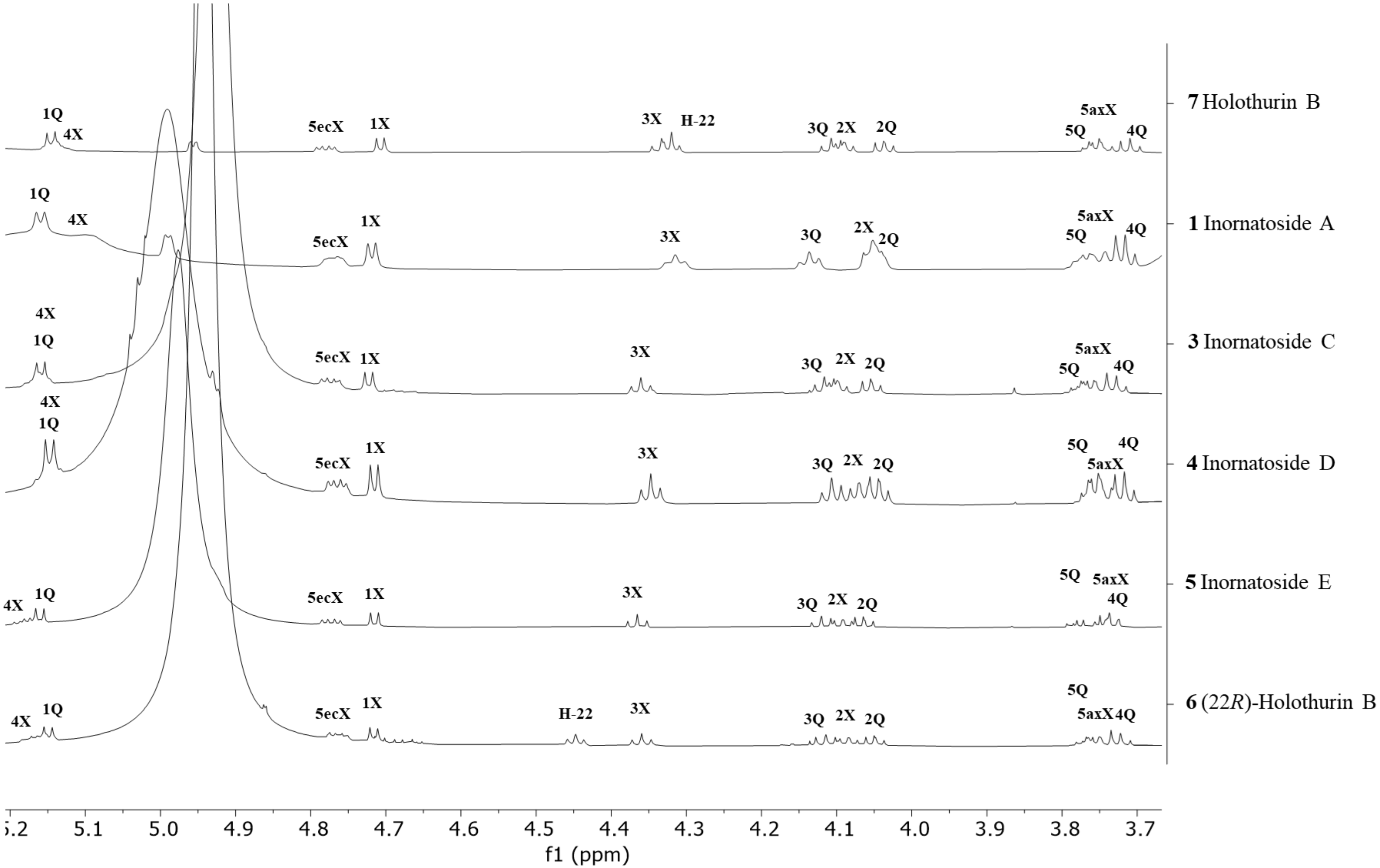

Figure S31. **^{1}H NMR spectra comparison: Signals of disaccharides (Q: quinovose, X: xylose) of compounds 1, 3-6 and 7 (700 MHz, Pyridine-d_5)**

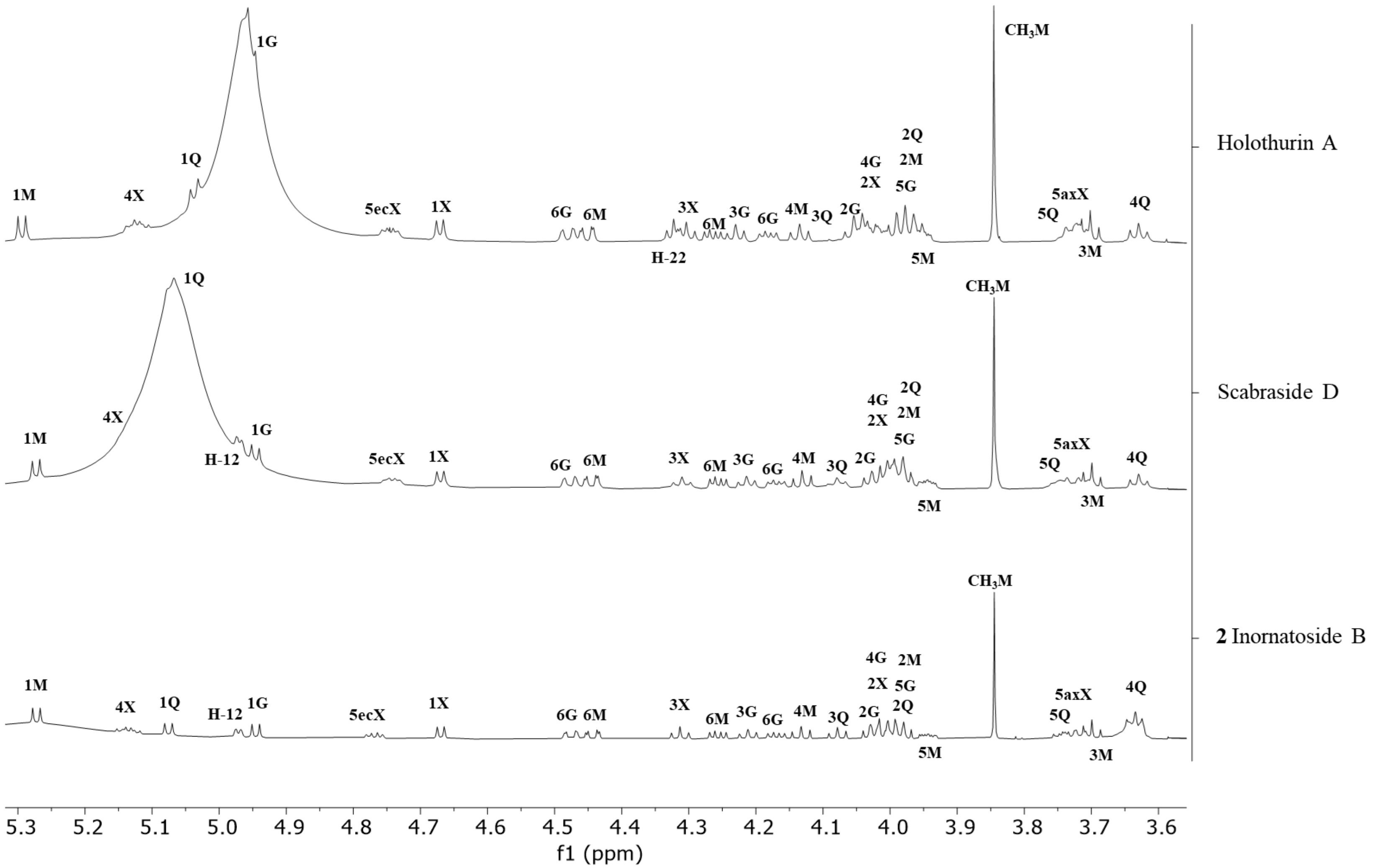

Figure S32. ^{1}H NMR spectra comparison: Signals of tetrasaccharides (Q: quinovose, X: xylose, G:glucose, M: methylglucose) of compounds 2, scabraside D and Holothurin A (700 MHz, Pyridine-*d*$_5$).

Table S1. ^{13}C and ^{1}H NMR data (*J* in Hz) for the aglycone moieties of known compounds (pyridine-d_5).[a,b]

	**Scabraside D**		**Holothurin B (7)**		**Holothurin A**	
	δ_C	δ_H	δ_C	δ_H	δ_C	δ_H
1_{ax}	36.4	1.39 ddd (13, 13, 4)	36.5	1.40 ddd (13, 13, 3)	36.4	1.39 ddd (13, 13, 4)
1_{eq}		1.81 brd (13)		1.81 ddd (13, 4, 3)		1.80 ddd (13, 3, 3)
2_{ax}	27.1	1.88 brddd (13, 13, 12)	27.1	1.90 dddd (14, 13, 12, 3)	27.1	1.88 brddd (13, 13, 13)
2_{eq}		2.08 brd (13)		2.09 brdd (14, 4)		2.08 brdd (13, 3)
3	88.8	3.13 dd (12, 4)	88.8	3.14 dd (12, 4)	88.8	3.11 dd (12, 4)
4	40.1	-	40.2	-	40.1	-
5	52.8	0.98 brd (11)	52.8	0.99 dd (12, 2)	52.7	0.98 brd (11)
6_{ax}	21.3	1.55 dddd (13, 13, 11, 2)	21.3	1.54 dddd (13, 13, 13, 2)	21.3	1.53 o
6_{ec}		1.74 o		1.73 o		1.70 o
7_{ax}	28.4	1.48 dddd (13, 13, 13, 4)	28.4	1.50 dddd (13, 13, 13, 4)	28.4	1.49
7_{eq}		1.74 o		1.75 o		1.75 o
8	41.0	3.35 brdd (13, 5)	41.0	3.34 brdd (13, 6)	41.0	3.34 brd (12)
9	154.1	-	153.9	-	153.9	-
10	39.8	-	39.8	-	39.8	-
11	115.7	5.58 brd (6)	115.6	5.60 dd (6, 2)	115.7	5.60 brs
12	71.5	4.97 brd (5)	71.6	4.96 dd (6, 2)	71.6	4.96 brd (5)
13	58.7	-	58.9	-	58.8	-
14	46.5	-	46.0	-	46.0	-
15_a	36.8	1.39 ddd (12, 12, 9)	36.9	1.39 ddd (12, 12, 9)	36.9	1.40 ddd (12, 12, 9)
15_b		1.81 dd (12, 9)		1.81 dd (12, 8)		1.82 dd (12, 8)
16_a	36.0	2.68 dd (15, 9)	35.7	2.96 dd (15, 9)	35.7	2.97 dd (15, 9)
16_b		2.33 ddd (15, 12, 9)		2.39 ddd (15, 12, 8)		2.40 ddd (15, 12, 8)
17	89.5	-	89.8	-	89.8	-
18	174.9	-	174.6	-	174.6	-
19	22.7	1.35 s	22.6	1.36 s	22.6	1.35 s
20	87.4	-	86.7	-	86.8	-
21	23.1	1.76 s	18.9	1.74 s	18.9	1.75 s
22	39.6	2H, 1.96 (o)	80.7	4.32 dd (7, 7)	80.7	4.32 dd (7, 7)
23_a	19.7	1.97 (o)	28.2	2.04 m	28.2	2.04 m
23_b		1.86 (o)		1.99 ddt (13, 8, 7)		1.99 m
24	45.3	2H 1.67 (o)	38.5	2H 1.60 t (7)	38.5	2H 1.60 t (8)
25	69.5	-	81.5	-	81.5	-
26	30.1	1.39 s	28.8	1.18 s	28.8	1.18 s
27	30.0	1.39 s	27.5	1.16 s	27.5	1.16 s
30	16.8	1.06 s	16.8	1.12 s	16.8	1.05 s
31	28.1	1.24 s	28.2	1.28 s	28.1	1.23 s
32	20.2	1.65 s	20.4	1.66 s	20.4	1.66 s
$\underline{CH_3}CO$						
$CH_3\underline{CO}$						
OH-12					-	7.81 s
OH-17	-	7.40 s			-	7.67 s

[a] Assignments were confirmed by ^{1}H-^{1}H-COSY, 1D and 2D-TOCSY, HSQC, HSQC-TOCSY and HMBC experiments. [b] o: overlapped with other signals.

Table S2. ^{13}C and ^{1}H NMR data (*J* in Hz) of the sugar chains of known compounds (pyridine-d_5).[a,b]

	**Scabraside D**		**Holothurin B (7)**		**Holothurin A**	
	δ_C	$\delta_{H\ (C\text{-}H)}$	δ_C	$\delta_{H\ (C\text{-}H)}$	δ_C	$\delta_{H\ (C\text{-}H)}$
1	105.4	4.67 d (7)	105.5	4.71 d (7)	105.3	4.67 d (7)
2	83.5	4.00 dd (7, 9)	83.3	4.09 dd (7, 9)	83.6	3.98 dd (7, 9)
3	76.0	4.31 dd (9, 9)	75.9	4.33 dd (9, 9)	75.8	4.30 dd (9, 9)
4	76.0	5.13 m	76.2	5.14 ddd (9, 9, 5)	75.7	5.12 ddd (9, 9, 6)
5_{ax}	64.5	3.72 dd (11, 9)	64.5	3.75 dd (12, 9)	64.5	3.72 dd (12, 9)
5_{ec}		4.74 dd (11, 5)		4.78 dd (12, 5)		4.75 dd (12, 6)
1’	105.5	5.07 o	106.1	5.15 d (8)	105.4	5.04 d (8)
2’	76.5	3.98 o	77.2	4.04 dd (8, 9)	76.4	3.97 dd (8, 9)
3’	76.1	4.08 dd (9, 9)	77.9	4.11 dd (9, 9)	75.9	4.05 dd (9, 9)
4’	87.5	3.63 dd (9, 9)	76.8	3.71 dd (9, 9)	87.3	3.63 dd (9, 9)
5’	71.8	3.75 o	73.6	3.75 dq (9, 6)	71.8	3.73 dq (9, 6)
6’	18.3	1.71 d (6)	18.8	1.65 d (6)	18.2	1.71 d (6)
1’’	105.1	4.95 d (8)			105.1	4.95 d (8)
2’’	73.9	4.03 dd (8, 9)			73.9	4.04 dd (8, 9)
3’’	88.2	4.21 dd (9, 9)			88.1	4.23 dd (9, 9)
4’’	70.0	4.01 o			69.9	4.02 o
5’’	78.1	4.00 o			78.1	4.01 o
6a’’	62.4	4.48 dd (12, 1)			62.2	4.48 dd (12, 2)
6b’’		4.17 dd (12, 6)				4.18 dd (12, 6)
1’’’	105.9	5.27 d (8)			105.8	5.29 d (8)
2’’’	75.2	3.98 dd (8, 9)			75.2	3.99 dd (8, 9)
3’’’	88.2	3.70 dd (9, 9)			88.2	3.70 dd (9, 9)
4’’’	70.7	4.13 dd (9, 9)			70.7	4.14 dd (9, 9)
5’’’	78.5	3.94 ddd (9, 6, 3)			78.5	3.95 ddd (9, 5, 2)
6a’’’	62.3	4.45 dd (12, 3)			62.2	4.45 dd (12, 2)
6b’’’		4.26 dd (12, 6)				4.26 dd (12, 5)
OCH_3	60.9	3.84 s			61.0	3.85 s

[a] Assignments were confirmed by ^{1}H-^{1}H-COSY, 2D-TOCSY, HSQC, HSQC-TOCSY and HMBC experiments.
[b] o: overlapped with other signals.
